# Spectral approaches to stress relaxation in epithelial monolayers

**DOI:** 10.1098/rspa.2024.0224

**Published:** 2024-09-11

**Authors:** Natasha Cowley, Christopher K. Revell, Emma Johns, Sarah Woolner, Oliver E. Jensen

**Affiliations:** 1 Department of Mathematics, University of Manchester, Oxford Road, Manchester M13 9PL, UK; 2 Manchester Cell Matrix Centre, School of Biological Sciences, University of Manchester, Oxford Road, Manchester M13 9PT, UK

**Keywords:** vertex model, stress relaxation, geometric stiffness

## Abstract

We investigate the viscoelastic relaxation to equilibrium of an isolated disordered planar epithelium described using the cell vertex model. In its standard form, the model is formulated as coupled evolution equations for the locations of vertices of confluent polygonal cells. Exploiting the model’s gradient-flow structure, we use singular-value decomposition to project modes of deformation of vertices onto modes of deformation of cells. Expressing the dynamics in terms of operators of a discrete calculus, we show how eigenmodes of discrete Laplacian operators (specified by constitutive assumptions related to dissipation and mechanical energy) provide a spatial basis for evolving fields, and demonstrate how the operators can incorporate approximations of conventional spatial derivatives. We relate the spectrum of relaxation times to the eigenvalues of the Laplacians, modified by corrections that account for the fact that the cell network (and therefore the Laplacians) evolve during relaxation to an equilibrium prestressed state, providing the monolayer with geometric stiffness. While dilational modes of the Laplacians capture rapid relaxation in some circumstances, showing diffusive dynamics, geometric stiffness is typically a dominant source of monolayer rigidity, as we illustrate for monolayers exposed to unsteady stretching deformations.

## 1 Introduction

The cell vertex model [1–4] provides a simple description of the mechanics of spatially disordered multicellular tissues, such as the epithelia that coat developing embryos, form our skin and line many internal organs [5]. The vertex model can capture elastic, viscous and plastic deformations [6,7], as well as more exotic features of spatially disordered glassy systems such as a jamming/unjamming transition [8]. The model is reasonably straightforward to implement computationally, at least in two dimensions, and it is readily integrated with models of biological processes such as cell division, cell signalling and cell motility [9]. The vertex model’s parsimony (in having a small number of material parameters) supports inference [10–14], providing the model with a degree of predictive power. Together, these features make the cell vertex model a popular tool in developmental biology, and in mechanobiology more generally, with simulations bringing valuable mechanical insights to the interpretation of experimental observations. Given its wide utility and application, it is helpful to understand and potentially exploit the model’s underlying mathematical structure.

It is well established that epithelial cells are sensitive to their mechanical environment [5]. For example, stretching of an epithelium stimulates cell division and reorients divisions along the axis of stretch, helping to dissipate stress and maintain epithelial homeostasis [15–19]. The responses of epithelial tissue to mechanical force are dynamic and adaptive; for example cell circularity following a uniaxial stretch can be restored close to pre-stretch levels in 90 min via actomyosin contraction [18]. Moreover, the force regimes experienced by epithelial tissues *in vivo* can vary significantly, from the fast changes seen upon wounding to the slower changes seen in the embryo during tissue morphogenesis. In order to investigate questions arising from these dynamic considerations, for example to benchmark deformation rates against intrinsic timescales of the monolayer, it is helpful to calculate the spectrum of viscoelastic relaxation times of a monolayer. These are determined not only by cell mechanical properties but also by the geometrical arrangements of cells within a monolayer. By incorporating the spatial disorder that is intrinsic to many natural tissues, the vertex model is particularly well suited to address the way in which geometry and topology together mediate multi-scale mechanical effects.

The cell vertex model rests on two primary assumptions. The first is that in two dimensions, a confluent cellular monolayer can be represented geometrically as a set of polygons that tile the plane. This abstraction allows cell shapes to be represented in terms of the locations of the vertices of the polygons. The second assumption is that a mechanical energy can be defined in terms of geometric features of the polygons. The principle of virtual work allows forces to be defined at vertices as gradients of the energy, and movement of vertices down this energy gradient (at a rate defined by a suitable model of dissipation) takes the system to the nearest equilibrium, possibly allowing cells to exchange neighbours (via so-called T1 transitions) in the process. In what follows, we adopt a variant of a simple but widely used energy, defined in terms of the area and perimeter of each cell, that has two dimensionless parameters; we also refine a common model of friction between cells and their substrate. This approach is undoubtedly an oversimplification of biological reality; nevertheless, a proper understanding of this foundational model is useful when introducing additional processes.

Development of a rigorous link between discrete (cell-scale) and continuous (tissue-scale) descriptions of multicellular tissues remains an open challenge, motivating a variety of upscaling approaches. Long-wave approximations assume disparate lengthscales and slow variation of properties over scales much longer than the size of cells, and have been conducted for the vertex model in one dimension [20,21] or in two dimensions via homogenization [22] or using probabilistic arguments [23]. Mean-field models are based either on a phenomenological macroscopic constitutive model [24–26] or rely on an assumption of regularity in the organization of cells [27–32]. Two studies in particular have emphasized the diffusive nature of the dynamics: coarse-graining of a one-dimensional analogue of the vertex model revealed diffusive dynamics in a Lagrangian frame of reference [20]; while a gradient flow of particles under an energy defined by areas of a tessellation was reduced to an (Eulerian) nonlinear diffusion equation [33]. In the present study, motivated in part by experiments [15–18] in which epithelia undergo an imposed stretch, we investigate a monolayer undergoing viscoelastic relaxation to an equilibrium; we remain in a discrete framework, while seeking points of contact with continuum descriptions. Our spectral perspective captures evolution across a wide range of lengthscales and timescales, retaining features that elude some upscaled models.

Building on studies of granular materials [34,35], we have demonstrated previously how the geometry of the cells themselves can be exploited in order to build operators of a discrete calculus [36,37], including spatial Laplacians defined over vertices and over cell centres. The eigenmodes of these operators provide bases for fields defined over a cell monolayer: exploiting Helmholtz–Hodge decomposition, we used this approach to represent the mechanical stress over a monolayer in terms of scalar potentials [36]. Low-order eigenmodes are sensitive to the shape of the monolayer as a whole, while high-order modes are sensitive to the shape of individual cells, providing a natural bridge between micro- and macroscopic features. (An alternative approach [38] uses a Laplacian that is weighted on the basis of a localized kernel function, rather than using the detailed structural information of individual cells.) The Laplacians identified in Jensen & Revell [36] provide a representation of scalar fields defined on cells and vertices and vector fields defined on cell edges and links between cell centres, and rely intrinsically on a primal network of cells and its dual triangulation. Below, we introduce complementary spatial Laplacians **ℒ**_ℱ_ and ℒ_ℱ_ that are suitable for discrete vector fields defined on vertices and scalar fields on faces, or vice versa, which do not require use of a dual network.

These discrete spatial operators provide a valuable framework for multi-scale analysis but are agnostic to the physical processes being modelled. However, the vertex model is a gradient flow on a manifold defined by both the choice of mechanical energy and the model for dissipation, implying a role for model-dependent operators. In the form of the vertex model that we consider, each cell is characterized by two scalar variables (area and perimeter), geometric attributes that are energy-conjugate to a pressure and a tension. The mapping between cell and vertex deformations is associated with the first variation of cell area and cell perimeter with respect to vertex locations. We will use these quantities (in a framework provided by singular-value decomposition (SVD)) to construct matrix operators that allow projection of the 2*N*_*v*_ degrees of freedom of the *N*_*v*_ vertices in ℝ^2^ onto the fewer 2*N*_*c*_ scalar variables defined over the *N*_*c*_ cells. Thereby, we construct a scalar Laplacian ${ℒ}_{c}^{\text{G}}$ and its vector equivalent ${𝓛}_{v}^{\text{G}}$ that combine physical parameters with geometric information. These model-dependent operators are constructed such that they have embedded within them the purely spatial Laplacians ℒ_ℱ_ and **ℒ**_ℱ_, as well as interactions with non-standard (and more non-local) spatial operators. Additional complexity arises from the fact that the network on which the operators are constructed evolves in time. This can arise in two ways: either through T1 transitions (ignored here), or from vertex motion associated with stress relaxation. Were the system defined on a static domain, one might expect the eigenvalues of ${ℒ}_{c}^{\text{G}}$ and ${𝓛}_{v}^{\text{G}}$ to determine the spectrum of relaxation times. This turns out typically not to be the case for monolayers in a jammed state: evolution of the prestressed network modifies relaxation times, endowing the monolayer with so-called geometric stiffness.

Spectra constructed from the full Hessian of the vertex model were computed by Tong *et al*. [39] in a study of monolayer viscoelasticity. A monolayer with *N*_*v*_ vertices has 2*N*_*v*_ degrees of freedom, a 2*N*_*v*_ × 2*N*_*v*_ Hessian and 2*N_v_* eigenvalues in its spectrum. Of this set, approximately one-quarter showed sensitivity to large variations in a parameter *Γ* (measuring resistance to perimeter change relative to area change), with the remaining modes being relatively insensitive [39]. We rationalize these observations by identifying a component of the spectrum that can be associated (for very large or small values of *Γ*) with a set of dilational modes of ${ℒ}_{c}^{\text{G}}$ and ${𝓛}_{v}^{\text{G}}$ ; we show how such modes have essentially diffusive dynamics. The remainder of the spectrum is strongly influenced by the monolayer’s geometric stiffness.

In the present study, we restrict attention primarily to monolayers in a jammed phase and ignore neighbour exchanges, extrusion and cell division. We formulate the vertex model in §2 using a framework that distinguishes cell-based from vertex-based evolution. We then use SVD in §3 to formally identify model-dependent Laplacian operators and to identify their contribution to the overall dynamics. This is illustrated via computation in §4. A gradient-flow perspective in §5 suggests connections with non-local aggregation–diffusion models. Technical details are provided in appendix A, where we use incidence matrices to perform key calculations.

## 2 The vertex model

### (a) Evolution equations for vertices and cells

In the cell vertex model, the evolution of a planar simply connected monolayer formed of *N*_*c*_ cells is simulated numerically by evolving the location of *N*_*v*_ vertices $r^{*}(t^{*})\equiv {\left(r_{1}^{*},\ldots ,r_{N_{v}}^{*}\right)}^{⊤}$ embedded in ℝ^2^ at time t*, giving the model 2*N*_*v*_ degrees of freedom (stars denote dimensional quantities). The vertices define a network of confluent polygonal cells. We assume all cells have the same material properties, and define the dimensional energy of cell *i* as $K_{A}^{*}U\left(A_{i}^{*}/A_{0}^{*}\right)+K_{L}^{*}U\left(L_{i}^{*}/L_{0}^{*}\right)$, for some positive constants $K_{A}^{*}$ and $K_{L}^{*}$. Here, $A_{i}^{*}$ and $L_{i}^{*}$ are the area and perimeter of cell *i*; $A_{0}^{*}$ and $L_{0}^{*}$ are a reference area and perimeter at which the relevant component of the energy is minimal. Accordingly, the dimensionless function 𝒰(*θ*) is assumed to be convex, satisfying 𝒰(1) = 𝒰′(1) = 0, 𝒰″(1) = 1 and 𝒰″(*θ*) > 0 for all *θ* > 0. We rescale lengths on $\sqrt{A_{0}^{*}}$ (so that $A_{i}^{*}=A_{0}^{*}A_{i},L_{i}^{*}=\sqrt{A_{0}^{*}}L_{i}$, etc.) and energy on $K_{A}^{*}$. Then, the dimensionless energy of cell *i* becomes $U_{i}(A_{i},L_{i})=U\left(A_{i}\right)+\Gamma L_{0}^{2}U(L_{i}/L_{0})$, where $L_{0}=L_{0}^{*}/\sqrt{A_{0}^{*}}$ and $\Gamma =\left(K_{L}^{*}/K_{A}^{*}L_{0}^{2}\right)$. *Γ* measures the relative energetic cost of cell perimeter change to area change. The pressure and tension of cell *i* are defined as *P*_*i*_ ≡ ∂*U*_*i*_*/*∂*A*_*i*_ = 𝒰′(*A*_*i*_) and *T*_*i*_ ≡ ∂*U*_*i*_*/*∂*L*_*i*_ = *ΓL*_*0*_𝒰′(*L*_*i*_*/L*_*0*_).

Taylor-expanding 𝒰 about *A*_*i*_ = 1 and *L*_*i*_ = *L*_0_ gives $U_{i}\approx \frac{1}{2}{\left(A_{i}-1\right)}^{2}+\frac{1}{2}\Gamma {\left(L_{i}-L_{0}\right)}^{2}$, recovering a commonly adopted expression for cell energy [2,40], for which *P*_*i*_ ≈ *A*_*i*_ − 1 and *T*_*i*_ ≈ *Γ* (*L*_*i*_ *− L*_*0*_). These linear pressure and tension relationships, if extrapolated, permit areas and perimeters to shrink to zero under finite energy change. To prevent this, we take 𝒰(*θ*) = *θ* (log *θ* – 1), giving

(2.1) $$P_{i}\left(A_{i}\right)=\log A_{i},\;T_{i}\left(L_{i}\right)=\Gamma L_{0}\log\left(L_{i}/L_{0}\right),$$

with $P_{i}^{\prime }\left(A_{i}\right)=1/A_{i}$ and $T_{i}^{\prime }\left(L_{i}\right)=\Gamma \left(L_{0}/L_{i}\right)$. The rigidity of an isolated cell is provided by tension *T*_*i*_ > 0 (with *L*_*i*_ > *L*_0_) balancing pressure *P*_*i*_ < 0 (with *A*_*i*_ < 1). Under (2.1), cell shrinkage requires greater mechanical energy than comparative cell expansion, while recovering the classical model for *A*_*i*_ near unity and *L*_*i*_ near *L*_0_ (so that for a perfect hexagon, *P*_*i*_ = 0 and *T*_*i*_ = 0 for *L*_0_ = 2 × 12^1/4^ ≈ 3.72). This formulation (2.1) accommodates heterogeneity of areas and perimeters across a monolayer, allowing significant deviations from *A*_*i*_ ≈ 1 and *L*_*i*_ ≈ *L*_0_.

Cells are characterized by two pairs of scalar attributes, making it convenient to group these quantities into vectors of length 2*N_c_*. We define g(*t*) to be the vector of cell pressures and tensions (so that g ≡ *P*_1_, …, $P_{N_{c}}$, *T*_1_, …, $T_{N_{c}}$)^⊤^), s(*t*) to be the corresponding vector of cell areas and perimeters (we write s(*t*) as shorthand for s(***r***(*t*))) and let s_0_ ≡ (1, 1, …, 1, *L*_0_, *L*_0_, …, *L*_0_)^⊤^ denote reference areas and perimeters. Pressures and tensions are related to areas and perimeters through the nonlinear function

(2.2) $$\text{g}=G\left(\text{s};{\text{s}}_{0},\Gamma \right),$$

that captures (2.1). The total mechanical energy of the monolayer $U=\underset{i}{\sum }U_{i}$ satisfies $\dot{U}={\text{g}}^{⊤}\dot{\text{s}}$ and *U*_s_ = g. Here, a dot denotes d/d*t* and the subscript s denotes a Fréchet derivative. Likewise, $\dot{\text{g}}=\text{G}\dot{\text{s}}$, where G is a matrix satisfying

(2.3) $$\text{G}=G_{\text{s}}\equiv \left(\begin{array}{cc} {\text{A}}_{c}^{-1} & 0\\ 0 & \Gamma L_{0}{\text{L}}_{c}^{-1} \end{array}\right),$$

where A_*c*_ = diag (*A*_*1*_, …, *A*_*Nc*_) and L_*c*_ = diag (*L*_1_, …, $L_{N_{c}}$). To explain notation, we use *i* = 1, …, *N*_*c*_ to sum over cells and *α* = 1, …, 2*N*_*c*_ to sum over pairs of attributes assigned to cells; *k* = 1, …*N*_*v*_ sums over vertices. We use a lower-case bold font to denote vectors in the physical plane ℝ^2^, lower-case sans serif to denote 2*N*_*c*_-vectors and sans serif italic to denote vectors or tensors with 2-vector-valued or tensor-valued components. We adopt matrix notation but use indices for clarity where necessary. ⊤ (or *T*) denotes a transpose with respect to coordinates *α* or *k* (or ℝ^2^).

Linearization around an equilibrium state (writing $\text{s}=\bar{\text{s}}+\hat{\text{s}}$, etc.) gives $\bar{\text{g}}=G(\bar{\text{s;}}\;{\text{s}}_{0},\Gamma )$ with perturbations satisfying $\hat{\text{g}}\;\approx \;\bar{\text{G}}\hat{\text{s}}$, using (2.3). The total energy, when expanded in terms of variations in areas and perimeters, is then $U\approx \underset{i}{\sum }\;{\bar{U}}_{i}+{\hat{\text{s}}}^{⊤}\bar{\text{g}}+\frac{1}{2}{\hat{\text{s}}}^{⊤}\bar{\text{G}}\;\hat{\text{s}}$. Accordingly, we define inner products for scalar-valued attributes u, v of cells, and vector-valued attributes ***u, v*** of vertices, as

(2.4a) $${\langle \text{u},\text{v}\rangle }_{\text{G}}\equiv {\text{u}}^{⊤}\text{Gv}\equiv \underset{\alpha ,\alpha^{\prime }}{\sum }u_{\alpha }G_{\alpha \alpha^{\prime }}v_{\alpha \prime }\;{\langle u,v\rangle }_{\text{E}}\equiv u^{⊤}\text{E}\cdot v\equiv \underset{k}{\sum }E_{k}u_{k}\cdot v_{k}.$$

Here, E = diag (*E*_1_, …, $E_{N_{v}}$) and *E*_*k*_ is an area assigned to each vertex, defined as the area of the triangle having vertices at the edge centroids neighbouring a given vertex (see figure 8*a* below). While G is the natural weighting for areas and perimeters, G^−1^ is the natural weighting for pressures and tensions, so that the quadratic contribution to the energy becomes

(2.4b) $$\frac{1}{2}\langle \hat{\text{s}},\hat{\text{s}}\rangle_{\bar{\text{G}}}=\frac{1}{2}\langle \hat{\text{g}},\hat{\text{g}}\rangle_{{\bar{\text{G}}}^{-1}}\equiv \frac{1}{2}{\hat{\text{g}}}^{\text{T}}{\bar{\text{G}}}^{-1}\hat{\text{g}}.$$

Some of the complexity of the vertex model originates in the nonlinear relationship between areas, perimeters and cell vertex locations. We capture this by defining **M**_*αk*_ = ∂*s*_*α*_*/*∂**r**_*k*_, or **M** = s_***r***_. The elements of ***M*** related to ∂*A*_*i*_/∂**r**_*k*_ can be visualized as the normal to the link between edge centroids adjacent to vertex *k* of cell *i*; those related to ∂*L*_*i*_*/*∂**r**_*k*_ correspond to unit vectors acting along the edges of cell *i* adjacent to vertex *k*, as explained in appendix Aa. Going to higher order, we can relate small changes in area and perimeter to vertex displacements by

(2.5) $$\delta s_{\alpha }=\underset{k}{\sum }M_{\alpha k}\cdot \delta r_{k}+\frac{1}{2}\underset{k,k^{\prime }}{\sum }\delta r_{k}\cdot M_{\alpha kk^{\prime }}^{\prime }\cdot \delta r_{k^{\prime }}+\ldots ,$$

where **M**′_*αkk*′_ ≡ ∂^2^*s*_*α*_/∂**r**_*k*_∂**r**_*k*_′ (see appendix Aa), and therefore

(2.6) $${\dot{s}}_{\alpha }=\sum_{k}M_{\alpha k}.{\dot{r}}_{k\text{,}}\;\text{i.e.}\;\dot{\text{s}}=M\cdot \dot{r}.$$

Here, we see a suggestion of ***M*** · acting analogously to a divergence operator, mapping a discrete (vector-valued) velocity field defined over vertices to (scalar-valued) area and perimeter changes defined over cells. This notion will be developed more formally below.

A common model of viscous dissipation (often chosen more for computational convenience than for mechanical realism) assigns a drag to each vertex, leading to the coupled evolution equations ${\dot{r}}_{k}=-\partial U/\partial r_{k}$ for *k* = 1, …, *N_v_* [2]. Here, time has been scaled so that the vertex drag coefficient is unity. We consider two refinements to this approach. First, in accounting for substrate drag alone, it is natural to assume that the drag force is proportional to a surface area of contact associated with each vertex. One natural choice for this area, defined solely in terms of the polygonal cell network, is *E*_*k*_, introduced in (2.4a). The monolayer is then assumed to evolve under $E_{k}{\dot{r}}_{k}=-\partial U/\partial r_{k}$. For a vertex in the interior of a monolayer, *E*_*k*_ is composed of three triangles, one lying in each adjacent cell; a vertex at the monolayer periphery, adjacent to one or two triangles, will therefore experience relatively lower drag. A further feature of this choice is that (1/*E*_*k*_) ∂*A*_*i*_/∂**r**_*k*_ has a direct interpretation as a discrete approximation of the spatial ∇ operator in ℝ^2^ (appendix Ab), from which a discrete Laplacian operator (in a Euclidean metric) can be constructed, acting on scalar fields defined over cells.

Movement of vertices down energy gradients can then be written (at vertex *k*, or over the whole monolayer) as

(2.7) $$E_{k}{\dot{r}}_{k}=-\sum_{\alpha }{\text{g}}_{\alpha }M_{\alpha k},\;\;\text{i.e.}\;\;\text{E}\dot{r}=-U_{r}\equiv -M^{⊤}\text{g},$$

with g satisfying (2.2). We can interpret (2.7) as a force balance on vertices, with drag on the left-hand side balancing elastic forces on the right; in a more global interpretation, (2.7) is a Darcy-type relation expressing the velocity field $\dot{\text{r}}$ as a gradient (in a sense defined below) of pressures and tensions.

A second model of dissipation is relevant when adhesion to the substrate is weak relative to dissipation internal to cells. The rate of dissipation owing to area and perimeter changes can be written (following (2.4a) and [6]) as

(2.8) $$\Phi \left(\dot{\text{s}}\right)={\langle \dot{\text{s}},\dot{\text{s}}\rangle }_{\text{H}}\text{where}\;\;\text{H}\equiv \left(\begin{array}{cc} \gamma_{A} & 0\\ 0 & \gamma_{L} \end{array}\right)⊗{\text{I}}_{N_{c}},$$

for some positive parameters *γ*_*A*_ and *γ*_*L*_. Here, ${\text{I}}_{N_{c}}$ is the *N*_*c*_ × *N*_*c*_ identity matrix. Equating the rate of change of mechanical energy with the rate of dissipation, the evolution in this case follows

(2.9) $$0=\dot{U}+\Phi ={\text{g}}^{⊤}\dot{\text{s}}+{\dot{\text{s}}}^{⊤}\text{H}\dot{\text{s}}=({\text{g}}^{⊤}M+{\dot{\text{s}}}^{⊤}\text{H}M)\cdot \dot{r},$$

indicating that the net force on the vertex (the quantity conjugate to the vertex velocity) is $-M^{⊤}\text{g}-M^{⊤}\text{H}M\cdot \dot{r}$. Combining this force with the substrate drag model (2.7) leads to the composite evolution equation

(2.10) $$\left(\text{E}⊗{\text{I}}_{2}+M^{⊤}\text{H}M\right)\cdot \dot{r}=-M^{⊤}\text{g}.$$

Two alternative non-local models of dissipation are discussed by Tong *et al*. [39], involving relative motion of neighbouring vertices and relative motion of neighbouring cell centres, but we do not pursue these here. Projecting (2.10) onto $\dot{r}$ shows that energy is dissipated as vertices move via

(2.11) $$\dot{U}=-\Phi -{\dot{r}}^{⊤}\cdot \text{E}\dot{r}\leq 0.$$

The evolution (2.2) and (2.10) can formally be written as the gradient flow $\dot{r}=-{\text{grad}}_{D}\;U$, using the dissipation operator in (2.10) as a metric; see appendix Ac. Here, the gradient is in a function space defined by the dissipation, and it is implemented formally as a 2*N*_*v*_ × 2*N*_*v*_ matrix that can be expected to be dense when H is non-zero.

To relate vertex dynamics to cell dynamics, we premultiply (2.10) by ***M*** · E^−1^ using (2.6), to give (2.2) plus

(2.12) $$\left({\text{l}}_{2N_{c}}+ℒ\text{H}\right)\dot{\text{s}}=-ℒ\text{g}\;\;\text{where}\;\;ℒ\equiv M\cdot {\text{E}}^{-1}M^{⊤}\equiv \left(\begin{array}{cc} {ℒ}_{A} & {ℒ}_{C}\\ {ℒ}_{C}^{⊤} & {ℒ}_{L} \end{array}\right).$$

The operator ℒ combines matrix multiplication (contracting over vertices) and a dot product (contracting over vectors in ℝ^2^) to construct a 2 × 2 matrix of *N*_*c*_ × *N*_*c*_ blocks with scalar components; diagonal elements ℒ_*A*_ and ℒ_*L*_ describe, respectively, squared area and perimeter changes of individual cells as a result of vertex displacements; off-diagonal elements ℒ_*C*_ and ${ℒ}_{C}^{⊤}$ describe the magnitude of area and perimeter changes arising from displacements of vertices shared by neighbouring cells. Equivalently, (2.12) can be expressed in terms of evolving pressures and tensions using

(2.13) $$\left({\text{I}}_{2N_{c}}+\text{G}ℒ\text{H}{\text{G}}^{-1}\right)\dot{\text{g}}=-\text{G}ℒ\text{g}.$$

Typically, pressures and tensions are coupled via the off-diagonal blocks ℒ_*C*_ and ${ℒ}_{C}^{⊤}$ of ℒ. For extreme values of *Γ*, however (see (2.3)), the operator Gℒ decouples into two components (appendix Ad) representing fast cell dilation and slow cell shear. For small (large) *Γ*, cell dilation is driven predominantly by pressure (tension) changes, and the relevant component of (2.13) is approximated by

(2.14a) $$\left(I+\gamma_{A}{\text{A}}_{c}^{-1}{ℒ}_{A}{\text{A}}_{\text{c}}\right)\dot{\text{P}}=-{\text{A}}_{c}^{-1}{ℒ}_{A}\text{P},\;\left(\Gamma \sim \gamma_{L}\ll 1\right),$$

(2.14b) $$\left(\text{I}+\gamma_{L}{\text{L}}_{\text{c}}^{-1}{ℒ}_{L}{\text{L}}_{\text{c}}\right)\dot{\text{T}}=-\Gamma L_{0}{\text{L}}_{\text{c}}^{-1}{ℒ}_{L}\text{T},\;\left(\Gamma \sim \gamma_{A}^{-1}\gg 1\right),$$

where P ≡ (*P*_1_, …, $P_{N_{c}}$)^⊤^ and T ≡ (*T*_1_, …, $T_{N_{c}}$)^⊤^; ~ denotes ‘scales like’. The operator $-{\text{A}}_{c}^{-1}{ℒ}_{A}$ in (2.14a), the first diagonal block of G*ℒ*, is a discrete approximation of the spatial ∇^2^ operator in ℝ^2^ (appendix Ab) when approximated on the polygonal grid defined by cells using the metrics E and G (see (2.3), (2.4a)), indicating that cell pressures can exhibit diffusive dynamics (mediated by viscous resistance to area change that is proportional to *γ*_*A*_). The corresponding operator $-{\text{L}}_{\text{c}}^{-1}{ℒ}_{\text{L}}$ in (2.14b) does not appear to have such a straightforward interpretation in terms of conventional derivatives.

In summary, (2.2) and (2.10) provide a full description of the dynamics (with 2*N*_*v*_ degrees of freedom), while (2.12) and (2.13) each describe the evolution of 2*N*_*c*_ cell-based variables, albeit depending on the 2*N*_*v*_ × 2*N*_*v*_ matrix ℒ. Despite a suggestion of diffusive dynamics in (2.13) and (2.14a,b), this interpretation is imperfect because the operator ℒ evolves with s and g via its dependence on ***r***; this can have a significant influence on the dynamics of the system.

### (b) Cell stress

While the model is formulated in terms of vertex locations, it is helpful to interpret its predictions in terms of cell stresses. At equilibrium, g and s are typically non-uniform (with g in the kernel of ***M***^⊤^), representing the fact that there can be residual stress (or prestress) in the equilibrium state of a disordered monolayer. The stress of cell *i* is ***σ***_*i*_ ≡ ***I***_2_*P*_*i*_ + (*L*_*i*_*T*_*i*_/ *A*_*i*_)***Q***_*i*_, where ***Q***_*i*_ is a shape tensor [6,40] defined in (A 9b) (appendix Aa) satisfying Tr(***Q***_*i*_) = 1; it quantifies the degree to which a cell is sheared. We can characterize cell stress through the magnitudes of the isotropic and deviatoric components of ***σ***_*i*_ [37,40], namely

(2.15) $$P_{\text{eff},i}=P_{i}+\frac{L_{i}T_{i}}{2A_{i}},\;\zeta_{i}=\frac{L_{i}T_{i}}{A_{i}}\sqrt{-\det\left(Q_{i}-\frac{1}{2}I_{2}\right)}.$$

An isolated hexagonal cell, for which *P*_eff, *i*_ = 0 and $Q_{i}=\frac{1}{2}I_{2}$, can still exhibit prestress at the level of individual vertices when *T_i_* > 0 (i.e. for *L*_0_ ≲ 3.72).

Defining ***W*** ≡ diag (I_2_, …, I_2_, ***Q***_1_, …, $Q_{N_{c}}$), (A 9a,b) shows that ***r* ⊗ *M***^⊤^ = ***W***s and hence, taking the trace,

(2.16) $$M\cdot r={\left(2A_{1},\ldots ,2A_{N_{c^{\prime }}}L_{1},\ldots ,L_{N_{c}}\right)}^{⊤};$$

(the factor of 2 is analogous to the identity ∇ · **x** = 2 in ℝ^2^), again hinting at the divergence-like nature of ***M*** ·. Applying ***r*** ⊗ to the force balance (2.10) leads to

(2.17) $$\frac{\text{d}}{\text{d}t}\{\frac{1}{2}r⊗\text{E}r+\frac{1}{2}{\text{s}}^{⊤}W\text{Hs}\}=-{\text{s}}^{⊤}W\text{g}\equiv -\sum_{i}A_{\text{i}}\sigma_{\text{i}}.$$

This sum over cells equates the elastic cell stress ***σ***_*i*_, integrated over the monolayer, with expressions measuring the rate of working of dissipative forces associated with vertex motion and changes in area and perimeter. At equilibrium, the left-hand side of (2.17) tends to zero, implying that the integrated stress also vanishes, although the stress in individual cells can be non-uniform.

As shown in eqn (2.3) of [6], the stiffness of a monolayer (under imposition of a small affine deformation with strain 𝔈) includes terms involving Tr(𝔈) and ***Q***_*i*_: 𝔈 (associated with changes in cell areas and perimeters, respectively) and ***B***_*i*_:𝔈 (where ***B***_*i*_ is a fourth-order tensor describing changes in ***Q**_i_* via reorientation of edges). The former terms contribute to the so-called material stiffness of the monolayer (by changing the energy directly), while the latter contributes to its geometric stiffness (providing stiffness without directly changing areas and perimeters). Similarly, tension-dependent terms associated with geometric stiffness appear in estimates of shear modulus under an assumed affine deformation (e.g. eqn (3.7) in [6]). We now consider the role of these different forms of stiffness in the viscoelastic relaxation of a monolayer, without making any assumptions about affinity.

### (c) Linearized dynamics

To better understand the relationship between the evolution of vertices and cells, we consider how the system relaxes to an equilibrium state. We write $\text{s}=\bar{\text{s}}\;+\;\hat{\text{s}}\left(t\right)$ (with ${\langle \hat{\text{s}},\;\hat{\text{s}}\rangle }_{\text{G}}\;\ll \;{\langle \bar{\text{s},}\bar{\text{s}}\rangle }_{\text{G}},$ etc.) to distinguish a steady state (with a bar) from decaying perturbations (with hats). In force balances, we neglect terms that are quadratic (or smaller) in hatted quantities. We Taylor-expand ***M*** with respect to **r**_*k*_ using (2.5) to obtain $\hat{M}={\bar{M}}^{\prime }\cdot \hat{r}$, so that ${\hat{M}}^{⊤}={\hat{r}}^{⊤}\cdot {\bar{M}}^{\prime ⊤}$. Steady states satisfy

(2.18) $$\bar{\text{g}}=G\left(\bar{\text{s}};{\text{s}}_{\text{0}},\Gamma \right),\;{\bar{M}}^{⊤}\bar{\text{g}}=0.$$

We then assume that all hatted quantities have time dependence *e^−λt^*, seeking the spectrum *λ*^(1)^, …, $\lambda^{\left(2N_{v}\right)}$ defined by possible values of *λ*; we suppress the superscript in what follows. Given the gradient-flow structure of the problem, we expect *λ* ∈ ℝ with *λ* ≥ 0. Evolving states satisfy, from (2.3), (2.6) and (2.10),

(2.19) $$\hat{\text{g}}=\bar{\text{G}}\hat{\text{s}},\;\hat{\text{s}}=\bar{M}\cdot \hat{r},\;-\lambda (\bar{\text{E}}⊗I_{2}+{\bar{M}}^{⊤}\text{H}\bar{M})\cdot \hat{r}=-{\bar{M}}^{⊤}\hat{\text{g}}\;-{\hat{M}}^{⊤}\;\bar{\text{g}}.$$

Thus, using $\hat{ℒ}=\hat{M}\;\cdot \;{\bar{\text{E}}}^{-1}\;{\bar{M}}^{⊤}+\bar{M}\cdot \;{\hat{\text{E}}}^{-1}\;{\bar{M}}^{⊤}+\bar{M}\cdot {\bar{\text{E}}}^{-1}\;{\hat{M}}^{⊤}$, the linearized dynamics of vertices and cells follows, respectively,

(2.20a) $$\lambda \left(\bar{\text{E}}⊗I_{2}+{\bar{M}}^{⊤}\text{H}\bar{M}\right)\;\cdot \hat{r}=\;\left({\bar{M}}^{⊤}\bar{\text{G}}\bar{M}+{\bar{\text{g}}}^{⊤}{\bar{M}}^{\prime }\right)\;\cdot \hat{r},$$

(2.20b) $$\lambda \left({\text{I}}_{2N_{c}}+\bar{ℒ}\text{H}\right)\hat{\text{s}}=\bar{ℒ}\bar{\text{G}}\hat{\text{s}}+\hat{ℒ}\bar{\text{g}}=\bar{ℒ}\bar{\text{G}}\hat{\text{s}}+\bar{M}\cdot \;{\bar{\text{E}}}^{-1}\left({\hat{r}}^{⊤}\cdot {\bar{M}}^{\prime ⊤}\right)\bar{\text{g}.}$$

We recognize ${\bar{M}}^{⊤}\bar{\text{G}}\bar{M}+\;{\bar{\text{g}}}^{⊤}\;{\bar{M}}^{\prime }$ in (2.20a) as the Hessian Ū _***r r***_, with terms representing, respectively, material stiffness and geometric stiffness [41,42]. Equivalently, expanding the energy to second order with respect to vertex locations using (2.5) gives

(2.21) $$U=\sum_{i}{\bar{U}}_{i}+{\hat{r}}^{⊤}\cdot {\bar{M}}^{⊤}\bar{\text{g}}+\frac{1}{2}{\hat{r}}^{⊤}\cdot [{\bar{M}}^{⊤}\bar{\text{G}}\bar{M}+{\bar{\text{g}}}^{⊤}\bar{M}\prime ]\cdot \hat{r}.$$

The evolution of cell pressures and areas is coupled to movement of vertices in (2.20b) via ${{\bar{M}}^{\prime }}^{⊤}\bar{\text{g}}$. A geometric interpretation of the associated forces is given in appendix Ae and steps needed to evaluate $\bar{M}$ and ${\bar{M}}^{\prime }$ are outlined in appendix Aa. The scalar and vector operators $\bar{L}$ and ${\bar{M}}^{⊤}\bar{\text{G}}\bar{M}$ · are suggestive of Laplacian operators, in a sense that we will qualify below. The relaxation rates *λ* appear as eigenvalues of the generalized eigenvalue problem (2.20a). The *λ*-spectrum was evaluated by Tong *et al*. [39] and Damavandi *et al*. [43] under periodic boundary conditions (using different models of dissipation). Here, we seek an understanding of the contributions of the Laplacian operators in (2.20a,b) to the dynamics of an isolated monolayer.

### (d) Simulations

We simulated the growth of isolated 100-cell monolayers, implementing a stochastic cell-division algorithm and T1 transitions to produce realizations of disordered monolayers for chosen values of *Γ* and *L*_0_; the monolayer periphery is force-free. Monolayers are successively relaxed to equilibrium for different parameter choices. We chose a disordered monolayer such that it maintained the same topology across the parameter space and did not contain any quadrilaterals at equilibrium. (Quadrilaterals can add the additional complexity of retaining localized prestress beyond the usual rigidity transition because their shape incompatibility does not allow them to relax their tension; in the jammed regime the exclusion of quadrilaterals makes a negligible difference to results.) We also simulated a symmetric 127-cell monolayer of hexagonal cells. In what follows, the parameters *Γ* and *L*_0_ are chosen primarily so that the monolayer is in a jammed state.

Simulation code is available as the VertexModel.jl package[44], previously developed for our past work in this area [36]. The DiscreteCalculus.jl package [45] was developed to aid the analysis in this paper. This package contains Julia code that creates operators discussed in this paper.

## 3 Singular-value decomposition

**Figure 1:**
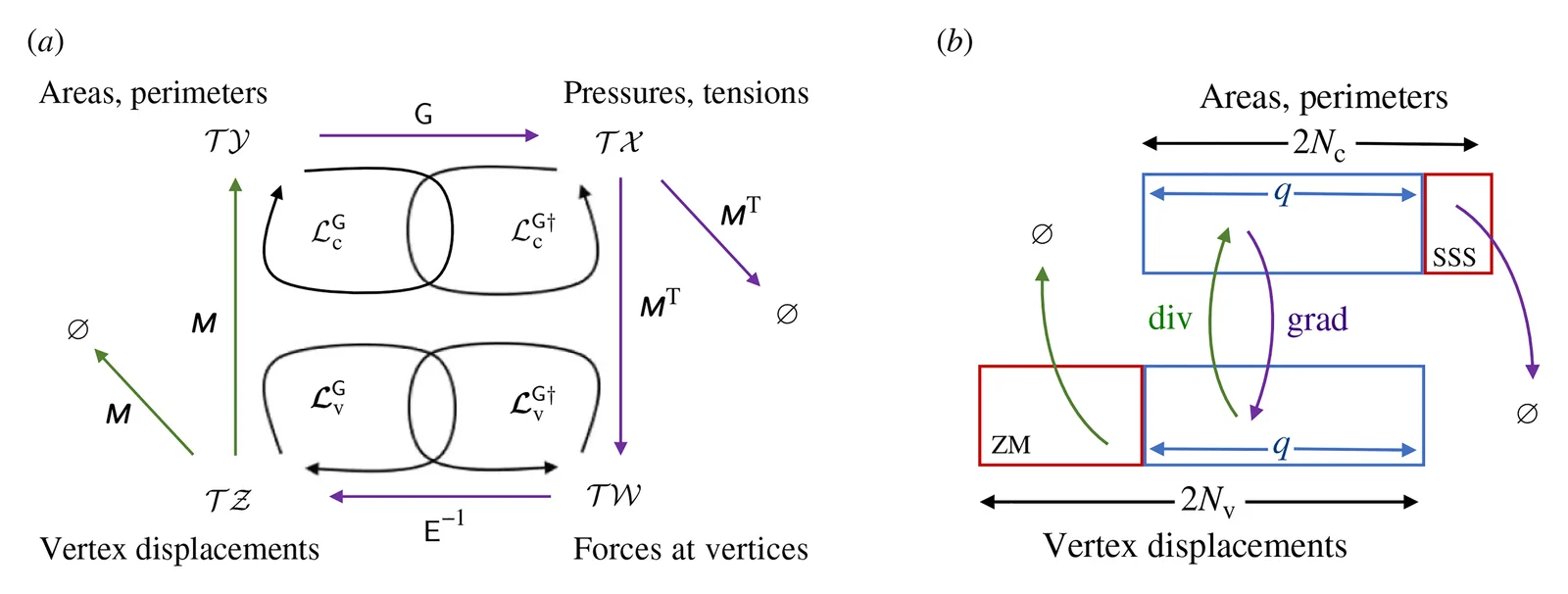
(*a*) A diagram summarizing the action of matrix operators ***M***, G and E. ***M*** has components that are vectors describing how cell areas and perimeters change under small vertex displacements; ‘vertex displacements’ describes the tangent space 𝒯 *Ƶ* of the state space *Ƶ*; ‘forces at vertices’ describes the dual space 𝒯 𝒲; ‘pressures, tensions’ sit in a space 𝒯 𝒳 that is dual to the tangent space 𝒯 𝒴 denoted ‘areas, perimeters.’ The operators ***M*** ⋅ and ***M***^⊤^ are singular with rank *q* < 2*N_c_* and therefore have non-trivial kernels mapping to ∅. Laplacians $L_{c}^{\text{G}}$ and ${𝓛}_{v}^{\text{G}}$ are defined in (3.4); dual operators are given in (3.9). (*b*) Kernels in 𝒯 *Ƶ* and 𝒯 𝒴 are shown as red boxes. Perturbations are mapped between these spaces by divergence ⟨***N***^⊤^, ⋅⟩_E_ and gradient ⟨***N***, ⋅⟩_G_ matrix operators. Elements of ker $\left({\bar{M}}^{⊤}\right)$ are denoted SSS; elements of $\text{ker}\left(\overset{¯}{M}\cdot \right)$ are denoted ZM. SSS, states of self-stress; ZM, zero modes.

In this section, in order to relate the full spectrum describing relaxation of vertices (with 2*N_v_* eigenvalues) to the potentially smaller system describing the evolution of 2*N_c_* scalars defined over cells, we reframe the problem using SVD. SVD provides a framework for understanding the action of the singular matrix operators ***M*** · and ***M***^⊤^, by identifying associated Laplacian operators (illustrated in figure 1) and enabling an assessment of their contribution to the overall dynamics.

Let $Z\subset {\mathbb{R}}^{N_{v}}\times {\mathbb{R}}^{2}$ be the (state) space of vertex locations and 𝒯 *Ƶ* be its tangent space. Let $Y\subset {\mathbb{R}}^{2N_{c}}$ be the space of areas and perimeters and 𝒯 𝒴 be its tangent space. The matrix operator ***M*** · (see (2.5)) maps from 𝒯 *Ƶ* to 𝒯 𝒴 (figure 1). We define q such that rank (***M***) ≡ *q* < 2N_*c*_ < 2*N*_*v*_. For a scalar-valued field Y ∈ 𝒯 𝒴, GY represents the associated variations in pressures and tensions; the norm ½ ⟨Y, Y⟩_G_ (see (2.4b)) measures the change in elastic energy associated with area and perimeter variation. GY sits in 𝒯 𝒳, the tangent space to the space of pressures and tensions, which is dual to 𝒯 𝒴 under (2.4a). For ***Z*** ∈ 𝒯 *Ƶ*, the norm ½⟨***Z, Z***⟩_E_ measures the dissipation associated with vertex motion. 𝒯 𝒲 (the space of force variations) is dual to 𝒯 *Ƶ* under (2.4a).

We then define ***N* = *M***E^−1^ so that ⟨***N***^⊤^, ⋅⟩_E_ maps from 𝒯 *Ƶ* to 𝒯 𝒴. Exploiting SVD [46], we can express ***N*** acting under the inner products (2.4a) as

(3.1) $$N=\sum_{p=1}^{q}\sigma_{p}{\text{Y}}_{p}Z_{p}^{⊤}\;\;\text{where}\;\;\left\{ \begin{array}{l} \langle Z_{p},Z_{p^{\prime }}\rangle_{\text{E}}=\delta_{pp^{\prime }}\;\;(1\leq p,p^{\prime }\leq 2N_{v}),\\ \left\langle {\text{Y}}_{p},{\text{Y}}_{p^{\prime }}\rangle_{\text{G}}=\delta_{pp^{\prime }}\;(1\leq p,p^{\prime }\leq 2N_{c}\right) \end{array}\right..$$

Here, $\left\{{\text{Y}}_{1}\ldots Y_{2N_{c}}\right\}$form an orthonormal basis of 𝒯 𝒴 and are the left-singular vectors of ***N***; $\left\{Z_{1},\ldots ,Z_{N_{v}}\right\}$ form an orthonormal basis of 𝒯 *Ƶ* and are the right-singular vectors of ***N***. The non-zero singular values of ***N*** are ordered as 0 < *σ*_1_ ≤ *σ*_2_ ≤ … ≤ *σ_q_* and the singular vectors are related via

(3.2a) $${\langle N^{⊤},Z_{p}\rangle }_{\text{E}}=\sigma_{p}{\text{Y}}_{p}\;(1\leq p\leq q),\;\langle N,{\text{Y}}_{p}\rangle_{\text{G}}=\sigma_{p}Z_{p}\;(1\leq p\leq q),$$

(3.2b) $${\langle N^{⊤},Z_{p}\rangle }_{\text{E}}=0\;(q+1\leq p\leq 2N_{v}),\;\langle N,{\text{Y}}_{p}\rangle_{\text{G}}=0\;(q+1\leq p\leq 2N_{c}).$$

From this, it follows that

(3.3) $${\langle N^{⊤},N^{⊤}\rangle }_{\text{E}}=N\text{E}\cdot N^{⊤}=\overset{q}{\underset{p=1}{\sum }}\sigma_{p}^{2}{\text{Y}}_{p}{\text{Y}}_{p}^{⊤},\;{\langle N,N\rangle }_{\text{G}}=N^{⊤}\text{G}N=\overset{q}{\underset{p=1}{\sum }}\sigma_{p}^{2}Z_{p}Z_{p}^{⊤}.$$

Thus, defining the vertex and cell operators

(3.4) $${ℒ}_{v}^{\text{G}}=N^{⊤}\text{G}N\text{E}\cdot ={\text{E}}^{-1}M^{⊤}\text{G}M\cdot ,\;L_{c}^{G}=N\text{E}\cdot N^{⊤}\text{G}=M{\text{E}}^{-1}\cdot M^{⊤}\text{G}\equiv L\text{G},$$

it follows that, for 1 ≤ *p* ≤ *p*, singular values can be related to eigenvalues of (3.4) via

(3.5) $${\langle N,{\langle N^{⊤},Z_{p}\rangle }_{\text{E}}\rangle }_{\text{G}}\equiv {𝓛}_{v}^{\text{G}}Z_{p}=\sigma_{p}^{2}Z_{p},\;{\langle N^{⊤},{\langle N,{\text{Y}}_{p}\rangle }_{\text{G}}\rangle }_{\text{E}}\equiv L_{c}^{\text{G}}{\text{Y}}_{\text{p}}=\sigma_{\text{p}}^{2}{\text{Y}}_{\text{p}}.$$

The operators ${𝓛}_{v}^{\text{G}}$ and $L_{c}^{\text{G}}$ are self-adjoint under the relevant inner products, because (taking transposes, for any fields $Z,\tilde{Z},\hat{\text{Y}},\text{Y}$)

(3.6a) $${\langle \tilde{Z},{𝓛}_{v}^{\text{G}}Z\rangle }_{\text{E}}={\tilde{Z}}^{⊤}\cdot M^{⊤}\text{G}M\cdot Z=Z^{⊤}\cdot M^{⊤}\text{G}M\cdot \tilde{Z}={\langle Z,{𝓛}_{v}^{\text{G}}\tilde{Z}\rangle }_{\text{E′}}$$

(3.6b) $${\langle \tilde{\text{Y}},L_{c}^{\text{G}}\text{Y}\rangle }_{\text{G}}={\tilde{\text{Y}}}^{⊤}\text{G}M{\text{E}}^{-1}\cdot M^{⊤}\text{GY}={\text{Y}}^{⊤}\text{G}M{\text{E}}^{-1}\cdot M^{⊤}\text{G}\tilde{\text{Y}}={\langle \text{Y},L_{c}^{\text{G}}\tilde{\text{Y}}\rangle }_{\text{G}}.$$

In addition, the operators are positive semi-definite because, for any ***Z***, Y,

(3.7) $${\langle Z,{𝓛}_{v}^{\text{G}}Z\rangle }_{\text{E}}={\langle {\langle N^{⊤},Z\rangle }_{\text{E}},{\langle N^{⊤},Z\rangle }_{\text{E}}\rangle }_{\text{G}}\geq 0,\;{\langle \text{Y},L_{c}^{G}\text{Y}\rangle }_{\text{E}}={\langle {\langle N,\text{Y}\rangle }_{\text{G}},{\langle N,\text{Y}\rangle }_{\text{G}}\rangle }_{\text{E}}\geq 0.$$

The operators also satisfy $N\text{E}\cdot {𝓛}_{v}^{\text{G}}={ℒ}_{c}^{\text{G}}N\text{E}.$ and $N^{⊤}\text{G}{ℒ}_{c}^{\text{G}}={𝓛}_{v}^{\text{G}}N^{⊤}\text{G}$. In summary, as illustrated in figure 1, the gradient operator ⟨***N***, ⋅ ⟩_G_ (the matrix operator E^−1^***M***^⊤^G) maps areas and perimeters to vertex displacements; the divergence operator ⟨ ***N***^⊤^, ⋅⟩_E_ (the matrix operator ***M*** ⋅) sums vertex displacements over cells to give changes in areas and perimeters. The combinations div ∘ grad and grad ∘ div in (3.4) form scalar (cell) and vector (vertex) Laplacians ${ℒ}_{c}^{\text{G}}$ and ${𝓛}_{v}^{\text{G}}$, respectively. The left- and right-singular vectors are the eigenvectors of ${ℒ}_{c}^{\text{G}}$ and ${𝓛}_{v}^{\text{G}}$, respectively; the two families of eigenmodes can be related to each other via (3.2a). The zero modes of the two operators (3.2) are not directly related to each other (for example, translation and rotation modes will be among the zero modes of ${𝓛}_{v}^{\text{G}}$ but do not have an analogue in ${ℒ}_{c}^{\text{G}}$). For a monolayer with symmetries, we can expect some singular values to be repeated.

Equivalently, we can also define Laplacians associated with dissipation owing to area and perimeter changes

(3.8) $${𝓛}_{v}^{\text{H}}=N^{⊤}\text{H}N\text{E}\cdot ={\text{E}}^{-1}M^{⊤}\text{H}M\cdot ,\;{ℒ}_{c}^{\text{H}}=N\text{E}\cdot N^{⊤}\text{H}=M{\text{E}}^{-1}\cdot M^{⊤}\text{H},$$

which again will share non-zero eigenvalues, and which satisfy $N\text{E}\cdot {𝓛}_{v}^{\text{H}}={ℒ}_{c}^{\text{H}}N\text{E}$⋅ and $N^{⊤}\text{H}{ℒ}_{c}^{\text{H}}={𝓛}_{v}^{\text{H}}N^{⊤}\text{H}$.

A dual formulation using $N^{†}\equiv M^{⊤}\text{G}=\sum_{p=1}^{q}\sigma_{p}W_{p}{\text{X}}_{p}^{⊤}$ maps pressure and tension variations X_*p*_ ∈ 𝒯 𝒳 to force variations ***W***_*p*_ ∈ 𝒯 𝒲, via the gradient ${\langle N^{†⊤},\cdot \rangle }_{{\text{G}}^{-1}}=M^{⊤}$ and divergence ${\langle N^{†},\cdot \rangle }_{{\text{E}}^{-1}}=\text{G}M{\text{E}}^{-1}$, yielding Laplacians acting, respectively, on forces and pressures plus tensions (see figure 1)

(3.9) $${𝓛}_{v}^{\text{G}†}=N^{†}{\text{G}}^{-1}N^{†⊤}{\text{E}}^{-1}\cdot =M^{⊤}\text{G}M{\text{E}}^{-1}\cdot ,\;{ℒ}_{c}^{\text{G}†}=N^{†⊤}{\text{E}}^{-1}\cdot N^{†}{\text{G}}^{-1}=\text{G}M{\text{E}}^{-1}\cdot M^{⊤}\equiv \text{G}ℒ.$$

The evolution equation for the monolayer (2.10) can then be written in terms of forces ***f*** (satisfying $\dot{f}=\text{E}\dot{r}$) as

(3.10a) $$\dot{f}+{ℒ}_{v}^{\text{H}†}\dot{f}=-{\langle N^{†⊤},\text{g}\rangle }_{{\text{G}}^{-1}},$$

again illustrating the gradient-flow structure of the evolution. Applying ${\langle N^{†},\cdot \rangle }_{{\text{E}}^{-1}}$ to (3.10a) recovers (2.12) in its dual form

(3.10b) $$\dot{\text{g}}+\text{G}{ℒ}_{c}^{\text{H}}{\text{G}}^{-1}\dot{\text{g}}=-{ℒ}_{c}^{\text{G}†}\text{g}.$$

Pressures and tensions evolve under the operator ${ℒ}_{c}^{\text{G}†}$, which has *q* degrees of freedom but which is determined by the larger number (2*N*_*v*_) of vertices. Equation (3.10a) generalizes to two dimensions the one-dimensional Lagrangian evolution equation derived in Fozard *et al*. [20]: the evolving force field (dual to the velocity field) balances a pressure (and tension) gradient plus a viscous term penalizing stretching; the associated kinematic relation (3.10b) suggests diffusive dynamics. However, we must again recognize that area and pressure changes can arise from dynamic changes in the operator ${ℒ}_{c}^{\text{G}†}$.

### (a) Spectral contributions of geometric and material stiffness

Having set the model in an SVD framework, we now identify the contributions of Laplacians to the overall dynamics. An equilibrium state (denoted with a bar) satisfies ${\bar{M}}^{⊤}\bar{\text{g}}=0$ (see (2.18)), placing ${\bar{\text{G}}}^{-1}\bar{\text{g}}$ in the kernel of ${\langle \bar{N},\cdot \rangle }_{\bar{\text{G}}}$ and making $\bar{\text{g}}$ a harmonic function with respect to ${ℒ}_{c}^{\bar{\text{G}}†}$. Thus

(3.11) $${\bar{\text{G}}}^{-1}\bar{\text{g}}=\sum_{p=q+1}^{2N_{c}}\gamma_{p}{\bar{\text{Y}}}_{p},$$

for some coefficients *γ_p_*. We require that q ≤ 2*N_c_* − 1, to allow the existence of at least one zero mode of ${\bar{ℒ}}_{c}^{\text{G}}$ (a state of self-stress, SSS) that can accommodate an equilibrium solution.

Consider small disturbances $\hat{r}$ and $\hat{\text{s}}=\langle \bar{N},\hat{r}\rangle$ to the equilibrium. We Taylor expand ***N*** so that $N=\bar{N}+{\langle {\bar{N}}^{\prime },\hat{r}\rangle }_{\bar{\text{E}}}+\cdots ,$, where $\bar{N}\prime ={\bar{\text{E}}}^{-1}M^{\prime }{\bar{\text{E}}}^{-1}$. Assuming dissipation via the substrate dominates that in the cells, (2.18) and (2.20b) become

(3.12a) $$0={\langle \bar{N},{\bar{\text{G}}}^{-1}\bar{\text{g}}\rangle }_{{\bar{\text{G}}}^{\prime }}$$

(3.12b) $$\dot{\hat{r}}=-{\bar{𝓛}}_{v}^{G}\hat{r}-{\langle {\langle \bar{N}\prime ,\hat{r}\rangle }_{\bar{E}},{\bar{G}}^{-1}\bar{\text{g}}\rangle }_{\bar{\text{G}}},$$

(3.12c) $$\dot{\hat{\text{s}}}=-{\bar{ℒ}}_{c}^{\text{G}}\hat{\text{s}}-{\langle {\bar{N}}^{T},{\langle {\langle \bar{N}\prime ,\hat{r}\rangle }_{\bar{\text{E}}},{\bar{\text{G}}}^{-1}\bar{\text{g}}\rangle }_{\bar{\text{G}}}\rangle }_{\bar{\text{E}}}.$$

We can map (3.12b) to (3.12c) by applying ${\langle {\bar{N}}^{⊤},\cdot \rangle }_{\bar{\text{E}}}$. Likewise, ${\langle \bar{N},\hat{\text{s}}\rangle }_{\bar{\text{G}}}={\bar{𝓛}}_{v}^{\text{G}}\hat{r}$, so that acting on (3.12c) with ${\langle \bar{N},\cdot \rangle }_{\bar{\text{G}}}$ recovers ${\bar{𝓛}}_{v}^{G}$ acting on (3.12b).

We assume that deformations can be captured in a basis provided by the left- and right-singular vectors, writing

(3.13) $$\hat{r}=\sum_{p=1}^{q}\alpha_{p}(t){\bar{Z}}_{p}+\sum_{p=q+1}^{2N_{v}}\beta_{p}(t){\bar{Z}}_{p},\;\hat{s}=\sum_{p=1}^{q}\sigma_{p}\alpha_{p}(t){\bar{\text{Y}}}_{p}.$$

The *q* modes in which vertex motions are coupled directly to area and perimeter changes have amplitudes *α*_*p*_; remaining modes with amplitudes *β*_*p*_ lie in the kernel of ${\langle {\bar{N}}^{⊤},\cdot \rangle }_{\bar{\text{E}}}$, representing vertex motions that have no direct impact on areas and perimeters. Defining

(3.14) $$D_{pp^{\prime }}={\langle {\bar{Z}}_{p},{\langle {\langle {\bar{N}}^{\prime },{\bar{Z}}_{p^{\prime }}\rangle }_{\bar{\text{E}}},{\bar{\text{G}}}^{-1}\bar{\text{g}}\rangle }_{\bar{\text{G}}}\rangle }_{\bar{\text{E}}},$$

and noting that $D_{pp^{\prime }}=D_{p^{\prime }p},$ we substitute (3.13) into (3.12b), project the dynamics onto individual modes and exploit orthogonality to recover

(3.15) $$(\begin{array}{c} \dot{\alpha }\\ \dot{\beta } \end{array})=-\left(\begin{array}{cc} {\text{D}}^{\alpha \alpha }+\text{diag}\left(\sigma_{p}^{2}\right) & {\text{D}}^{\alpha \beta }\\ {\text{D}}^{\alpha \beta } & {\text{D}}^{\beta \beta } \end{array}\right)(\begin{array}{c} \alpha \\ \beta \end{array}),$$

where $(\alpha ,\beta )=\left(\alpha_{1},\ldots ,\alpha_{q},\beta_{q+1},\ldots ,\beta_{N_{v}}\right),\;D_{pp^{\prime }}^{\alpha \alpha }=D_{pp^{\prime }}$ for $1\leq p,p^{\prime }\leq q,\;D_{pp^{\prime }}^{\alpha \beta }=D_{pp^{\prime }}\;\text{for}\;1\leq p\leq q<p^{\prime }<2N_{v}\;\text{and}\;D_{pp^{\prime }}^{\beta \beta }=D_{pp^{\prime }}\;\text{for}\;q\leq p,p^{\prime }<2N_{v}$. Eigenvalues *λ*^(*s*)^ of the matrix in (3.15) ($\tilde{\text{D}}$, say) recover the full spectrum of relaxation rates of the vertices. Orthonormal eigenvectors (*α*^(*s*)^, *β*^(*s*)^)^⊤^, With (3.13) recover the associated spatial modes ${\hat{r}}^{\left(s\right)}e^{-\lambda^{\left(s\right)}t}$, which are in turn orthonormal under ⟨⋅, ⋅⟩_Ē_: specifically, using (3.1),

(3.16) $$\begin{array}{l} {\langle {\hat{r}}^{\left(s\right)},{\hat{r}}^{\left(s^{\prime }\right)}\rangle }_{\bar{\text{E}}}={\langle \underset{p}{\sum }\alpha_{p}^{\left(s\right)}Z_{p}+\underset{p}{\sum }\beta_{p}^{\left(s\right)}Z_{p\prime }\underset{p^{\prime }}{\sum }\alpha_{p^{\prime }}^{\left(s^{\prime }\right)}Z_{p^{\prime }}+\underset{p^{\prime }}{\sum }\beta_{p^{\prime }}^{\left(s^{\prime }\right)}Z_{p^{\prime }}\rangle }_{\bar{\text{E}}}\\ \;=\underset{p}{\sum }\alpha_{p}^{\left(s\right)}\alpha_{p}^{\left(s^{\prime }\right)}+\underset{p}{\sum }\beta_{p}^{\left(s\right)}\beta_{p}^{\left(s^{\prime }\right)}=\delta_{ss^{\prime }}. \end{array}$$

Inserting ${\hat{r}}^{\left(s\right)}e^{-\lambda^{\left(s\right)}t}$ into (3.12a,b) and then contracting with ${\langle {\hat{r}}^{\left(s\right)},\cdot \rangle }_{\bar{\text{E}}}$ gives

(3.17) $$\lambda^{\left(s\right)}=\underset{p}{\sum }\sigma_{p}^{2}{\left[\alpha_{p}^{\left(s\right)}\right]}^{2}+\underset{p,p^{\prime }}{\sum }\alpha_{p}^{\left(s\right)}D_{pp^{\prime }}\alpha_{p^{\prime }}^{\left(s\right)}+\underset{p,p^{\prime }}{\sum }\beta_{p}^{\left(s\right)}D_{pp^{\prime }}\beta_{p^{\prime }}^{\left(s\right)}.$$

(Equation 3.17) allows us to evaluate how the decay rate of a given mode is built from eigenvalues of the Laplacian operators (the terms involving $\sigma_{p}^{2}$) and from interaction with the prestressed equilibrium state (the terms involving D, which capture evolution of the operators). The area and perimeter changes associated with eigenvector ${\hat{r}}^{\left(s\right)}$ are specified by non-zero components in *α*^(*s*)^; the associated first-order energy change ${\hat{\text{s}}}^{⊤}\bar{\text{g}}$ is proportional to $\sum_{p^{\prime }=1}^{q}\sigma_{p^{\prime }}\alpha_{p^{\prime }}^{\left(s\right)}{\langle {\bar{\text{Y}}}_{p^{\prime }},{\bar{\text{G}}}^{-1}\bar{\text{g}}\rangle }_{{\bar{\text{G}}}^{\prime }}\lambda^{\left(s\right)}$ in (3.17) gives the second variation of the energy in (2.21) for mode *s*.

In summary, reformulation of the problem using SVD has revealed the equilibrium Laplacian operators ${\bar{ℒ}}_{c}^{\text{G}}$ and ${\bar{𝓛}}_{c}^{\text{G}}$ that drive the dynamics, while providing spatial bases that connect vertex dynamics to patterns of cell area and perimeter change. Using (3.17), we can now assess the relative contributions of material and geometric stiffness to the dynamics, with the former driven by Laplacian operators and the latter by the coupling between vertex motions and the equilibrium prestress, which perturbs these operators.

## 4 Results

**Figure 2:**
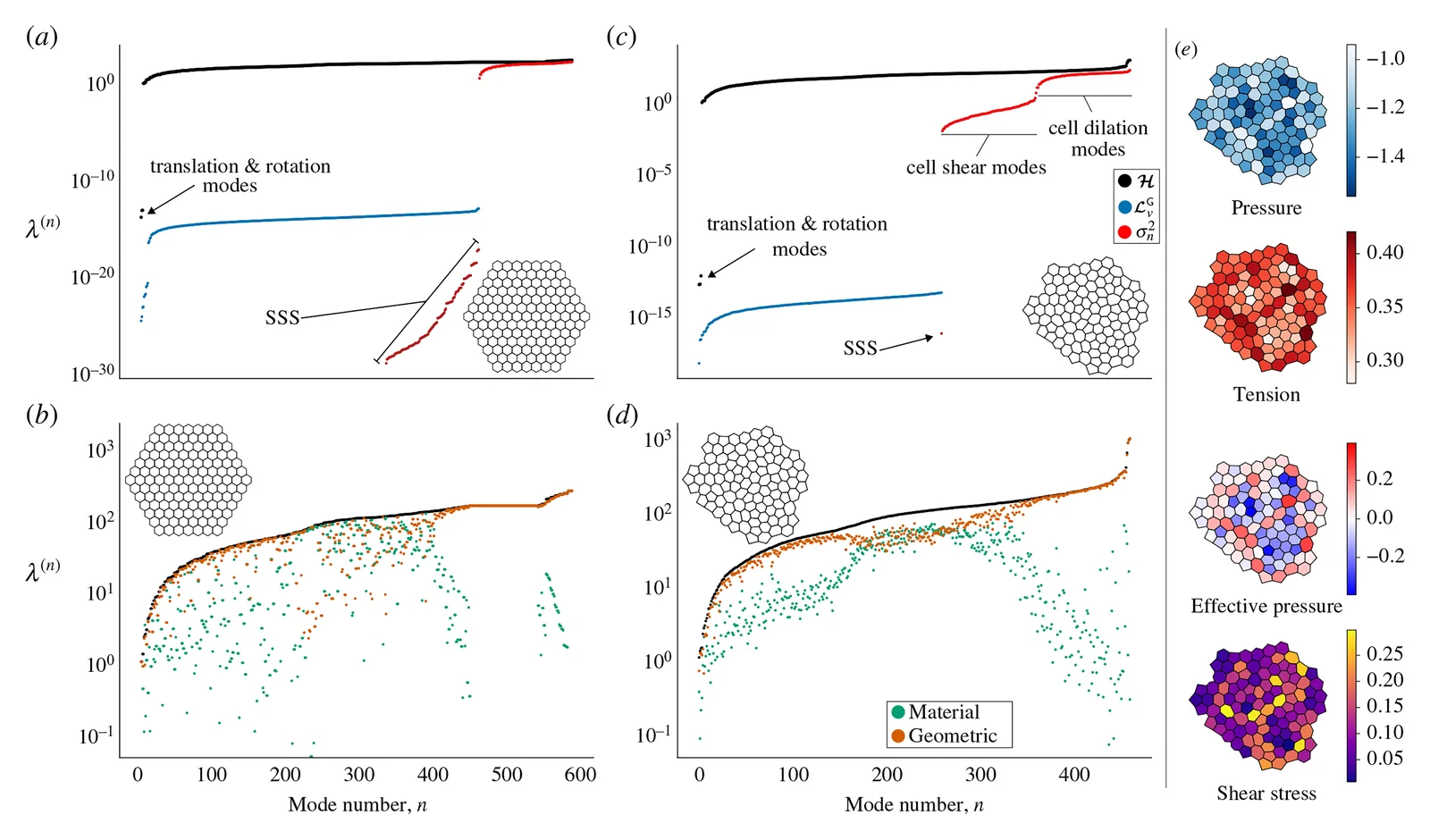
Relaxation spectra of a symmetric and a disordered monolayer. (*a,b*) Spectra for a symmetric configuration of *N_c_* = 127 hexagonal cells, as depicted in the inset; (*c,d*) spectra for a disordered monolayer of *N_c_* = 100 cells; *Γ* = 0.5, *L*_0_ = 1, *γ*_*A*_ = *γ*_*L*_ = 0 in both cases. (*e*) Shows the corresponding pressure, tension and isotropic and deviatoric cell stress components *P*_eff, *i*_ and ζ_*i*_ in (2.15) of the equilibrium disordered monolayer. (*a,c*) Compare the eigenvalues *λ*^(*n*)^, *n* = 1, …, 2*N*_*v*_ of the Hessian operator (2.20a, black) with the 2*N*_*v*_ eigenvalues of the vertex Laplacian (3.4, blue) and the *q* non-zero eigenvalues of the cell Laplacian (3.4, red); *N*_*v*_ = 294 and *q* = *N*_*c*_ in (*a*) and *N*_*v*_ = 228 and *q* = 2*N*_*c*_ − 1 = 199 in (*c*); zero modes of ${\bar{𝓛}}_{v}^{\text{G}}$ have *λ*^(*n*)^ < 10^−10^; translation and rotation modes of the Hessian are indicated; the *N_c_* zero modes in (*a*) and single zero mode in (*c*) of ${\bar{ℒ}}_{c}^{\text{G}}$are indicated as SSS; cell-shear and cell-dilation modes are indicated in (*c*). (*b, d*) The Hessian spectrum on an enlarged scale, excluding the translation and rotation zero modes, decomposed using (3.17) into contributions from material stiffness (green, derived from ${ℒ}_{c}^{\text{G}}$) and geometric stiffness (orange, derived from prestress).

Spectra for a symmetric and a disordered monolayer (each in a jammed state, with *Γ*= 0.5, *L*_0_ = 1) are presented in figure 2. It is helpful to examine these examples together, to identify common features and some important differences. The q non-zero eigenvalues of the Laplacian operators ${\bar{ℒ}}_{c}^{\text{G}}$ and ${\bar{𝓛}}_{c}^{\text{G}}$ overlap: *q* = 2*N*_*c*_ − 1 (*q* = *N*_*c*_) for the disordered (symmetric) monolayer. For the disordered monolayer, ${\bar{ℒ}}_{c}^{\text{G}}$ therefore has a single zero eigenvalue corresponding to the SSS associated with the equilibrium, shown in figure 2*e*; ${\bar{𝓛}}_{v}^{\text{G}}\;\text{has}\;2N_{v}-q=2N_{v}-2N_{c}+1$ has 2*N*_*v*_ − *q* = 2*N*_*v*_ − 2*N*_*c*_ + 1 zero modes (for which *λ* < 10^−10^ in figure 2*c*). The symmetric monolayer possesses 2*N*_*v*_ − *N*_*c*_ zero modes of ${\bar{𝓛}}_{v}^{\text{G}}$ and *N*_*c*_ zero modes of ${\bar{ℒ}}_{c}^{G}$ (figure 2*a*). We describe the two branches of ${\bar{ℒ}}_{c}^{\text{G}}$ evident in figure 2*c* as cell-shear and cell-dilation modes, for reasons given below; for the symmetric monolayer, the cell-shear modes have (effectively) zero eigenvalue, becoming SSS modes. However, the full spectrum of the Hessian (computed using (2.20a) and via projection of the dynamics onto right-singular vectors (3.17)) hides this underlying structure, with geometric stiffness dominating the bulk of the spectrum (figure 2*b,d*). This stiffness is provided by the prestress in the monolayer. Heterogeneities of the isotropic and deviatoric components of cell stress (figure 2*e*) reflect heterogeneities in cell pressures and tensions. By contrast, cells in the hexagonal array have zero cell stress, although prestress is still provided by uniform cell pressures and tensions. Geometric stiffness exploits sums of tensions and differences of pressures across edges (appendix Ae), suggesting that tensions are the dominant source of geometric stiffness in the symmetric case. In both examples, the Hessian has three zero modes (representing two translations and a rotation; figure 2*a,c*). A plateau in the spectrum of the hexagonal array (figure 2*b*) reflects a symmetry in the associated modes (which involve motion of internal vertices; a similar plateau was reported in Tong *et al*. [39]).

The relative contributions of material and geometric stiffness were reported for the vertex model in Damavandi *et al*. [43] in terms of the density of states of the Hessian and its two component matrices (see (2.20a)), averaged over realizations, assuming periodic boundary conditions. Our results (figure 2*b,d*) are consistent with these findings, but offer a more finely resolved picture for isolated monolayers, using instead the decomposition (3.17).

**Figure 3:**
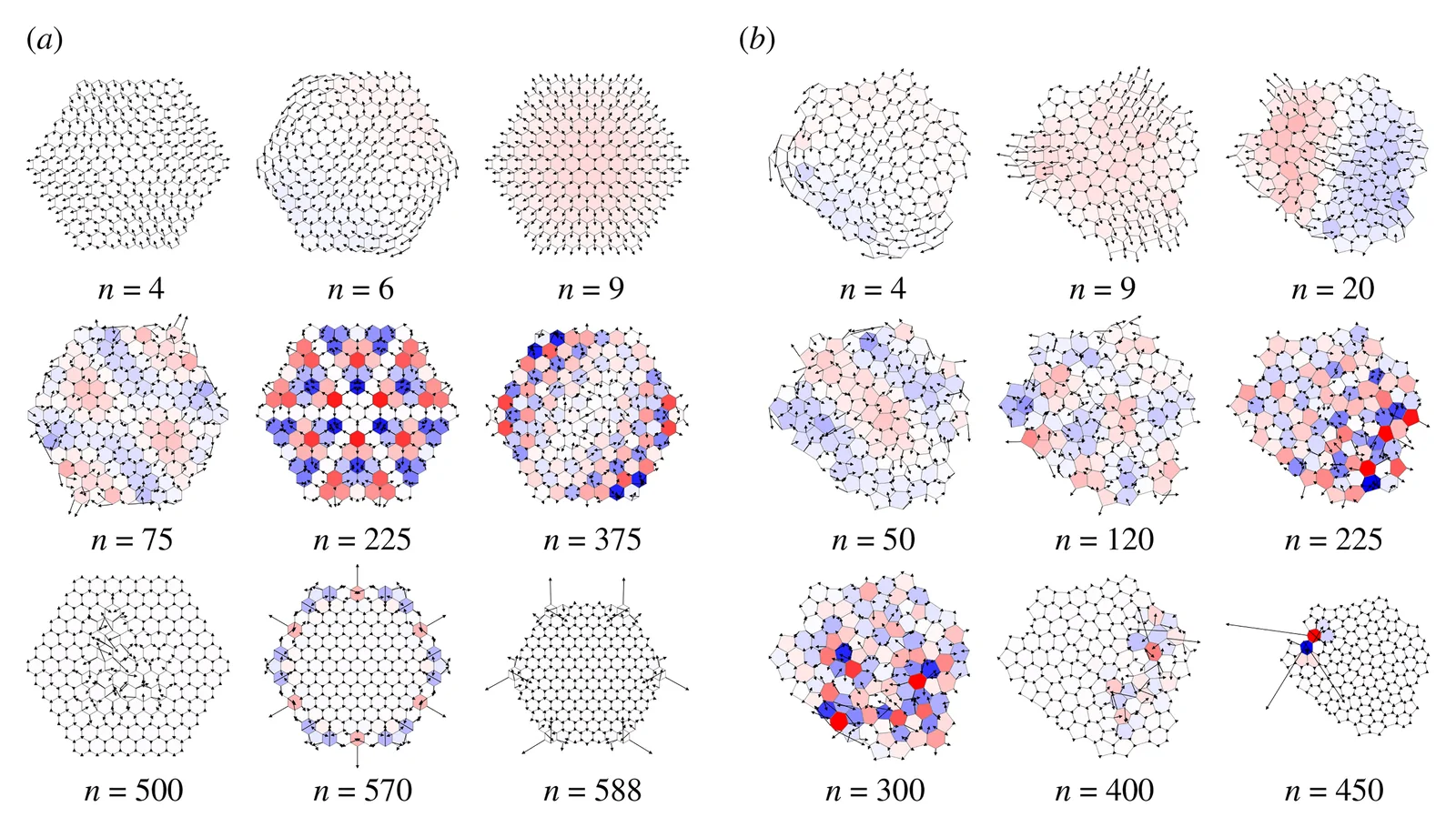
A selection of eigenmodes of the Hessian of (*a*) the hexagonal monolayer and (*b*) the disordered monolayer shown in figure 2. The corresponding eigenvalues are ranked by index *n*; arrows show vertex displacements, colours show corresponding cell area variations, with red and blue indicating variations of opposite signs.

Eigenmodes of the Hessians (figure 3) of the disordered and symmetric monolayers involve vertex displacements that may lead to changes in cell area and perimeter; displacements are typically non-affine. Moving along the eigenvalue spectrum, the slowest decaying modes involve deformations over lengthscales comparable with the size of the monolayer; modes with shorter lengthscales decay more quickly. For the symmetric monolayer, mode 500 (of 592) illustrates an internal deformation appearing in the plateau of the spectrum shown in figure 2*a*, for which there is no change in cell area; modes 570 and 588 illustrate how the upturn at the right-hand end of the spectrum is associated with modes confined to external cells. For the disordered monolayer, rapidly decaying modes (such as 400 and 450, of 456) are spatially localized.

**Figure 4:**
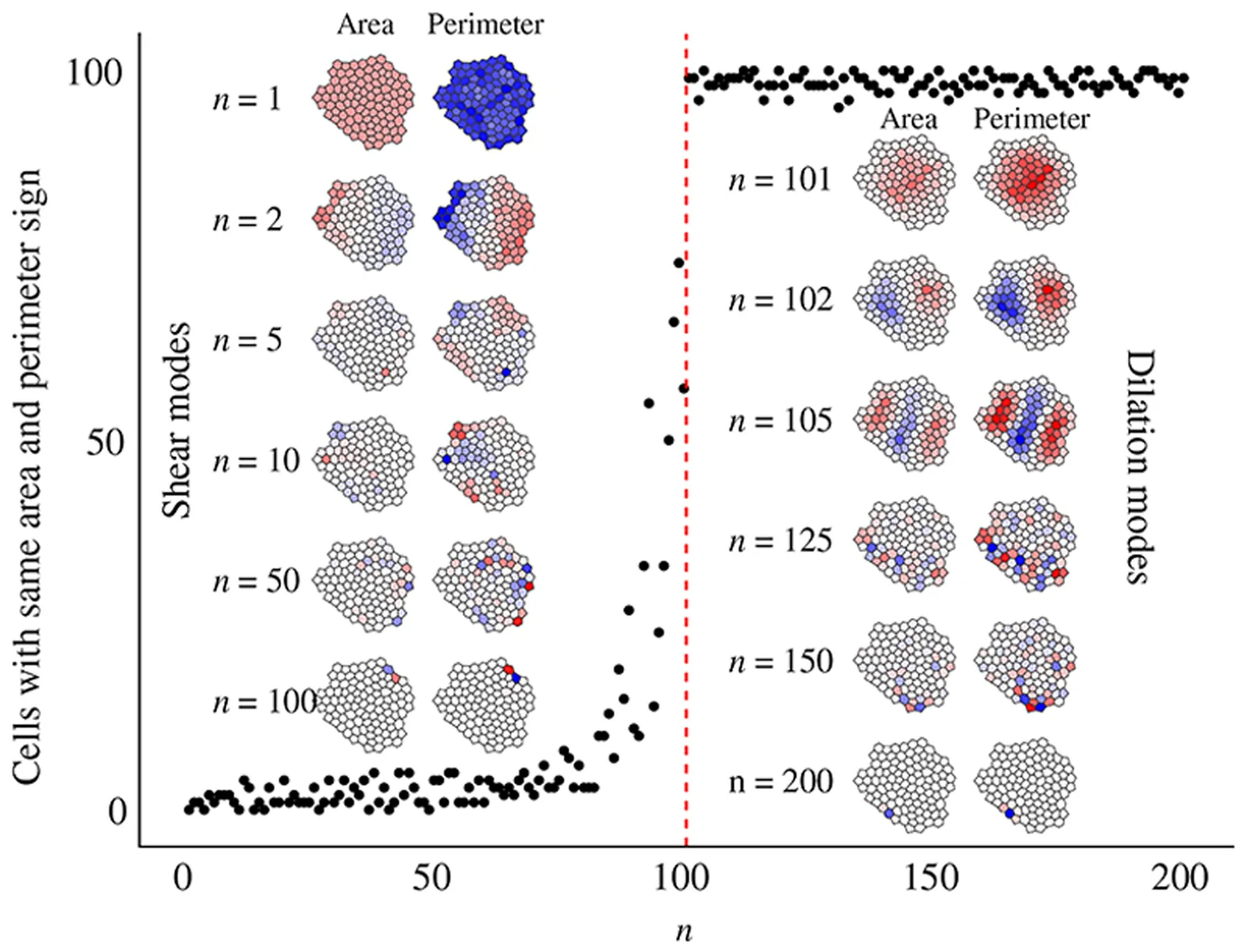
Insets show eigenmodes of the cell Laplacian ${\bar{ℒ}}_{c}^{\text{G}}$, for the 100-cell disordered monolayer shown in figure 2, with mode numbers as indicated; colours indicate patterns of relative area and perimeter variation (red and blue show opposite signs). Shear (dilation) modes (left (right)) are defined to have area and perimeter values of opposite (the same) parity; the categorization is clear for the majority of modes, as demonstrated in the scatterplot, which counts the number of cells that have the same sign for their area and perimeter values. Mode *n* = 1 is the SSS.

The spatial structure of the eigenmodes of the underlying scalar Laplacian ${\bar{ℒ}}_{c}^{\text{G}}$ for the disordered monolayer are illustrated in figure 4. These form two distinct groups, representing cell shear and cell dilation. Dilation modes relax faster than shear modes (figure 2*c*), with pressure and tension perturbations having the same sign; shear modes typically have pressure and tension perturbations of opposite sign (figure 4). Labelling these modes with 1 ≤ *n* ≤ 2*N*_*c*_, mode *n* = 1, with eigenvalue zero (the SSS), has GY_1_ proportional to $\bar{\text{g}}$ (see (3.11)). Low-order shear modes (e.g. n = 2, 5) have lengthscales comparable with the whole monolayer, with area and perimeter perturbations of opposite sign. As the mode number increases towards N_*c*_, the eigenmodes become increasingly localized. Long-wave cell-dilation modes appear for modes *N*_*c*_ + 1 = 101, *N_c_* + 2 = 102, this time with area and perimeter perturbations having the same sign. Again, these modes become increasingly localized as the mode number increases towards 2*N*_*c*_. The corresponding eigenmodes of ${\bar{𝓛}}_{v}^{\text{G}}$ (giving more fine-grained vertex displacements) are readily determined via (3.2a).

**Figure 5:**
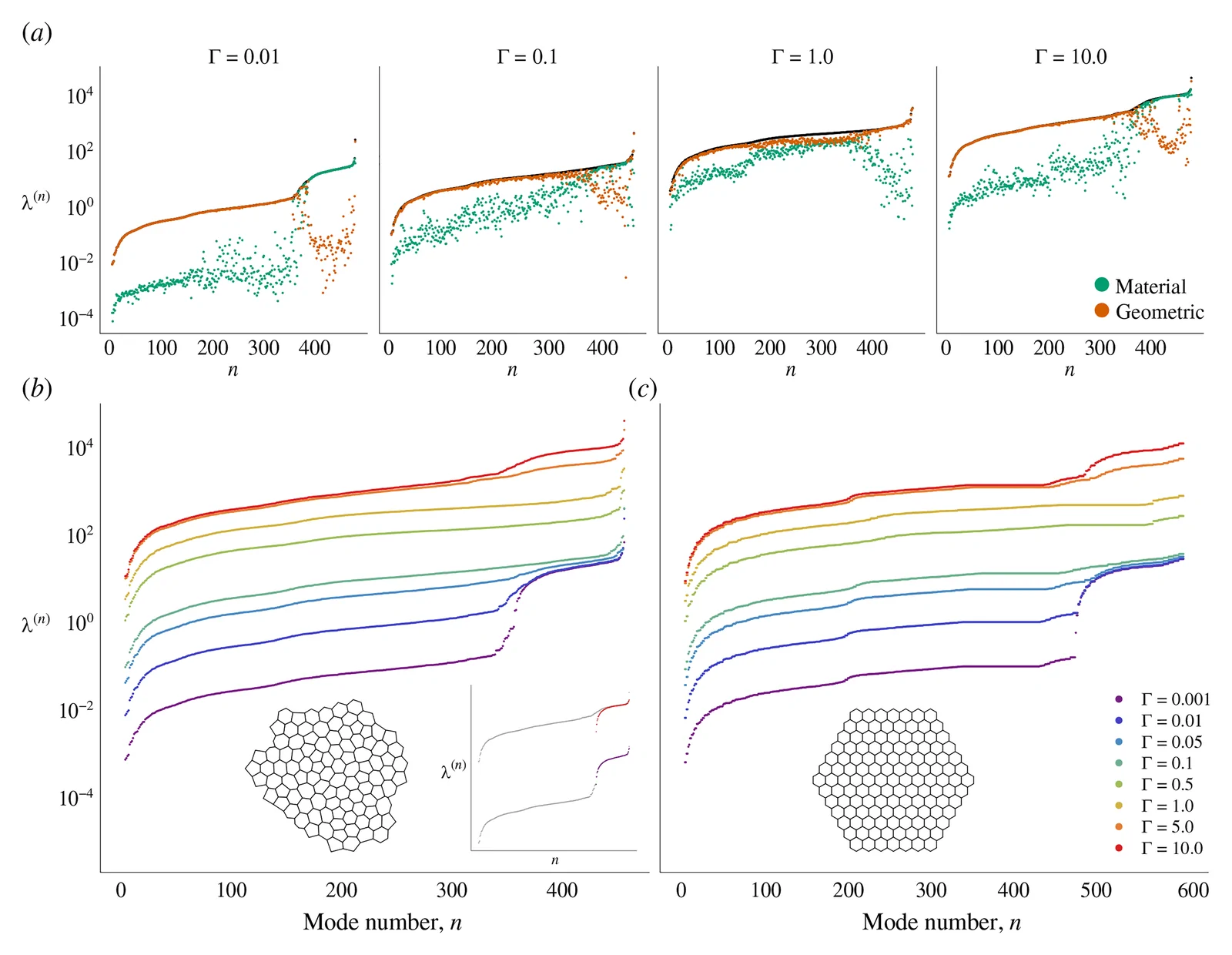
Impact of variation of the stiffness parameter *Γ* on relaxation spectra. (*a*) Spectra for *L*_0_ = 1 and *Γ* = 0.01, 0.1, 1, 10 for realizations of disordered monolayers with *N*_*c*_ = 100, *N*_*v*_ = 228, showing contributions of geometric (orange) and material (green) stiffness to the full Hessian (black; translation and rotation modes are not shown). (*b*) Cell-dilation modes collapse for small *Γ* (blue, purple); the remainder of the spectrum collapses for large *Γ* (red, orange). The inset shows how the spectra of ${\text{A}}_{c}^{-1}{ℒ}_{A}$ for *Γ* = 0.001 (purple) and $\Gamma L_{0}{\text{L}}_{c}^{-1}{ℒ}_{L}$ for *Γ* = 10 (red) predict the most rapidly decaying component of the full spectrum (grey). (*c*) The same as (*b*) but for a monolayer of hexagons with *N*_*c*_ = 127, *N*_*v*_ = 294.

**Figure 6:**
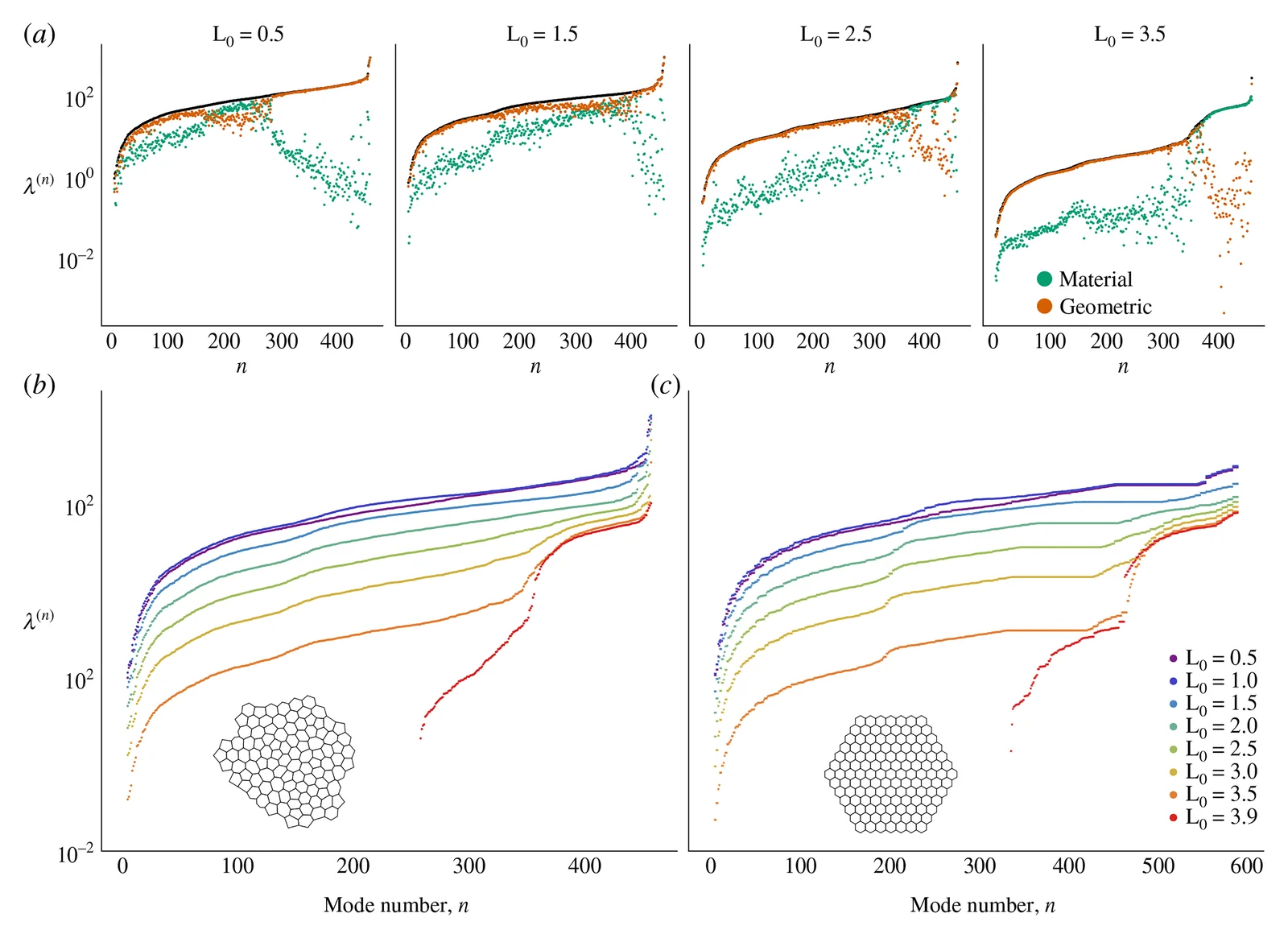
Impact of variation of the preferred perimeter *L*_0_ on relaxation spectra. (*a*) Spectra for *Γ* = 0.5 and *L*_0_ = 0.5, 1.5, 2.5, 3.5 for realizations of disordered monolayers with *N*_*c*_ = 100, *N*_*v*_ = 228, showing contributions of geometric (orange) and material (green) stiffness (translation and rotation modes are not shown). The case *L*_0_ = 0.5, *Γ* = 1 is shown in figure 2*d*. (*b*) Cell-dilation modes collapse (orange, red) as *L*_0_ approaches the critical value for the rigidity transition. After the rigidity transition (red), the Hessian spectrum completely collapses to the cell Laplacian spectrum and has 2*N*_*c*_ non-zero eigenmodes.(*c*) The same as (*b*) but for a monolayer of hexagons with *N*_*c*_ = 127, *N*_*v*_ = 294.

It is known [43] that near the jamming transition, ${\bar{ℒ}}_{c}^{\text{G}}$ (in our notation) may directly determine some modes of the Hessian. To establish more generally when this may arise, we computed spectra for different values of *Γ* and *L*_0_ (figures 5 and 6). For extreme values of *Γ*, the *N*_*c*_ cell-dilation modes capture the most rapidly decaying component of the spectrum (figure 5*a*), indicating that this component of the dynamics can be described by (2.14a) (with *γ*_*A*_ = 0 and *Γ* ≪ 1) or (2.14b) (with *γ*_*L*_ = 0 and *Γ* ≫ 1). This is further demonstrated by the inset to figure 5*b*, which shows how the most rapidly decaying modes of the full spectrum are resolved by the discrete approximation of ∇^2^ (appendix Ab) when *Γ* is small. (By contrast, cell-shear modes do not appear to play such a dominant role in determining the Hessian.) Thus, for *Γ* = 10 (figure 5*a*), tension dominates pressure and cells are close to their target perimeter: dilation modes determine the fastest segment of spectrum via (2.14b) with eigenvalues scaling with *Γ*; remaining modes are dominated by geometric stiffness, but show insensitivity to *Γ* (figure 5*b*). For *Γ* = 0.01 (figure 5*a*), pressure dominates tension and cells are close to their target area: cell-dilation modes determine the fastest segment of spectrum via (2.14a), with eigenvalues of order unity (showing *Γ*-independence in figure 5*b*); remaining modes are dominated by geometric stiffness, scaling with (small) *Γ*. For intermediate *Γ* (e.g. *Γ* = 1), with a balance between tension and pressure, geometric stiffness influences the entire spectrum. A similar picture emerges for the symmetric monolayer (figure 5*c*). In this instance, we find that the blocks of ℒ in equation (2.12) satisfy ${ℒ}_{L}={ℒ}_{C}^{⊤}{ℒ}_{A}^{-1}{ℒ}_{C}$ and ${ℒ}_{A}={ℒ}_{C}{ℒ}_{L}^{-1}{ℒ}_{C}^{⊤}$, ensuring that shear modes have zero eigenvalue (appendix Ad).

Increasing *L*_0_ towards a critical value reduces cell tensions towards zero and takes the jammed monolayer to an unjammed state. The approach to this transition is illustrated in figure 6*a*. At *L*_0_ = 3.5, the dilation modes are evident, but the eigenvalues of the remaining modes (deriving stiffness from prestress, particularly via tensions) drop substantially in magnitude, with only (slow) shear modes remaining once *L*_0_ = 3.9 (figure 6*b*). This is indicative of a switch from a jammed to an unjammed state. The transition takes place at values of *L*_0_ broadly consistent with prior studies using similar constitutive models (e.g. [39]). A similar loss of geometric stiffness is evident for the symmetric monolayer (figure 6*c*).

**Figure 7:**
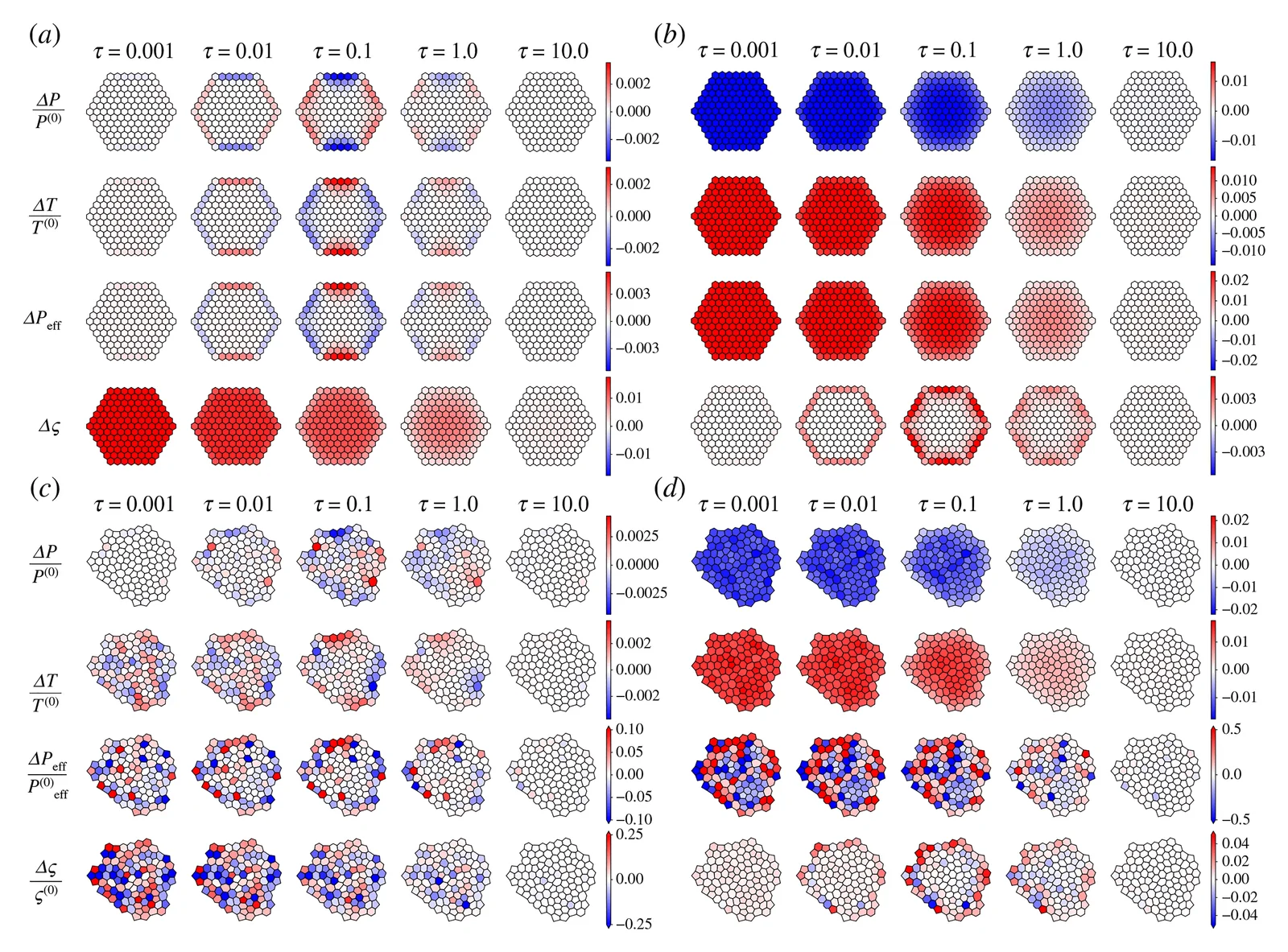
Changes in cell pressure, tension, isotropic stress *P*_eff_ and shear stress *ζ* (2.15) immediately after 1% uniaxial (*a,c*) and biaxial (*b,d*) stretch imposed over five timescales *τ* to the hexagonal (*a,b*) and disordered (*c,d*) monolayers shown in figure 2*c,d*. Most cases show relative changes (i.e. relative changes in magnitude) using normalizations as indicated; absolute changes in *P*_eff_ and *ζ* are reported for hexagonal cells. Since *P*_*i*_ < 0 (*T*_*i*_ > 0) because *A*_*i*_ < 1 (*L*_*i*_ > *L*_0_), an increase in area (perimeter) under stretch leads to a reduction (increase) in the magnitude of *P*_*i*_ (*T*_*i*_). Subsequent relaxation of pressure and tension for *τ* = 0.1 is illustrated for disordered cells in the electronic supplementary material, video S1 (uniaxial) and S2 (biaxial).

Returning to the baseline hexagonal and disordered monolayers illustrated in figure 2, we imposed a 1% stretch on each monolayer over a time interval *τ*, implemented as described in appendix Af. Stretch is modelled via a viscous drag imposed on vertices; the amplitude is too small to induce neighbour exchanges, and the monolayer relaxes to its original equilibrium after the stretch terminates. The five chosen rates 1/*τ* span the eigenvalue spectra. Considering first the response of the hexagonal monolayer (figure 7*a*), fast uniaxial stretch generates a shear-stress response that is distributed uniformly across the monolayer, becoming weaker for slower stretch. At intermediate stretch rates, however, peripheral cells show a different response to those in the bulk: the shear-stress response is weakened, with peripheral cells along the top and bottom boundaries (aligned with the main axis of stretch) showing evidence of expansion and those along the remaining boundaries showing evidence of compression. By contrast, fast biaxial loading of the hexagonal monolayer (figure 7*b*) generates uniform cell expansion, as reflected in the isotropic stress *P*_eff_; however, at intermediate loading rates, peripheral cells experience shear stress that appears to be associated with a reduction in the magnitude of the *P*_eff_ response.

Similar features are seen in the disordered monolayer, although these are modified by its intrinsic heterogeneity. The shear-stress response to rapid uniaxial stretching (figure 7*c*) is distributed across the monolayer; slower stretching weakens this response but induces expansion and compression of peripheral cells, although both patterns are heterogeneous. Rapid biaxial stretching (figure 7*d*) generates uniform cell expansion but no appreciable shear stress; again, at intermediate stretching rates, the changes to pressures and tensions in peripheral cells are weaker than those in the interior, although the peripheral cells now experience some shear stress. The electronic supplementary material, videos S1 and S2 [47] show how pressures and tensions relax after uni- and biaxial stretch; in the latter case, a largely homogeneous disturbance relaxes quickly, leaving slower evolution of a more heterogeneous pattern. In both examples, very slow loading (*τ* = 10) is too weak to elicit a strong response. In summary, figure 7 shows how patterns of geometric changes (reflected in pressures and tensions) can differ significantly from patterns of stress changes, showing sensitivity to the mode and rate of deformation; furthermore, cells at the monolayer periphery can experience deformations and stresses that are distinct from those in the interior.

## 5 Discussion

Despite being relatively straightforward to formulate and to simulate, the cell vertex model exemplifies many of the outstanding challenges in multi-scale modelling, particularly concerning the relationship between micro- and macroscopic mechanics of cellular materials. Here, we have used spectral techniques to investigate the relaxation of a multicellular material to an equilibrium state, as may arise after an imposed perturbation to a biological tissue. This approach is valuable in capturing deformations across all lengthscales, avoiding any assumptions of spatial or statistical regularity. Even in the presence of symmetry, the dynamics can be quite distinct from that expected from conventional continuum models.

We introduced three variations to the standard vertex model, by (i) modifying dissipation to account for the size of the region adjacent to each cell vertex undergoing drag as it slides over a substrate, (ii) incorporating viscous resistance to changes in cell area and perimeter, and (iii) introducing a mechanical energy that incorporates nonlinear pressure–area and tension–length relations, relaxing the requirement implicit in some conventional models that cell areas and perimeters should be everywhere close to preferred values. These three features influence metric matrices *E, H* and *G*, respectively, that appear in discrete differential operators within the model, and therefore influence stress relaxation. While most of our qualitative observations are insensitive to detailed modelling assumptions, (i) and (iii) were chosen specifically to highlight how the operator driving cell-dilation modes when cell bulk stiffness dominates cortical stiffness $-{\text{A}}_{c}^{-1}{ℒ}_{A}$ in (2.14a)) approximates the conventional spatial operator ∇^2^ (appendix Ab).

The operator governing cell-dilation modes when cortical stiffness dominates bulk stiffness $\left(-{\text{L}}_{c}^{-1}{ℒ}_{L}\right)$ is a discrete spatial second derivative with a more non-local structure. At a practical level, we found that the main impact of introducing *E*_*k*_ in (2.7), compared with the classical model with *E*_*k*_ = 1, is to scale the overall relaxation time of the monolayer by a factor roughly proportional to $\sum_{k=1}^{N_{v}}E_{k}/N_{v}$.

Using the monolayer as a network on which discrete differential operators can be constructed, we have clarified the relationship between the gradient-flow structure of the vertex model and generalized gradient and divergence operators (figure 1) that are used to build Laplacian operators. While these model-dependent operators incorporate the spatial operators $-{\text{A}}_{c}^{-1}{ℒ}_{A}$ and $-{\text{L}}_{c}^{-1}{ℒ}_{L}$ mentioned above, the generalized operators built from the matrix ***M*** and its transpose have a more abstract structure, mapping between vertex displacements (in ℝ^2^) and tangent spaces 𝒯 𝒴 and 𝒯 𝒳 that hold pairs of scalar attributes per cell (respectively, area and perimeter variations, and pressure and tension variations). Because ***M*** ⋅ and ***M***^⊤^ are singular (to accommodate an equilibrium SSS, and zero modes for which vertex motions do not change areas and perimeters), SVD provides a natural framework with which to formally establish properties of the Laplacian operators ${ℒ}_{c}^{\text{G}}$ and ${𝓛}_{v}^{\text{G}}$ and their duals ${ℒ}_{c}^{\text{G}†}$ and ${𝓛}_{v}^{\text{G}†}$, which underpin the model; their actions are illustrated in figure 1*a*.

Despite suggesting predominantly diffusive dynamics, the Laplacian operators evolve as the monolayer deforms, to the extent that geometric stiffness typically dominates material stiffness (figures 2, 5 and 6). Prestress in an equilibrium state can therefore have an important influence on the rate at which disturbances decay. A consistent exception is the manner in which the cell-dilation modes of ${ℒ}_{c}^{\text{G}}$ (involving area or perimeter changes, which directly change the mechanical energy of individual cells) capture the fastest *N*_*c*_ modes of the Hessian for extreme values of *Γ* (figure 5).

While our primary interest is in monolayers in a jammed state, the model illustrates how rigidity is lost through an increase in *L*_0_ (figure 6) via the well-studied jamming/unjamming transition [8], with cell-dilation modes (which exhibit resistance to area change, but not to shear) contributing primarily to monolayer stiffness past the unjamming transition. Rigidity can also be lost through reduction in *Γ* towards zero (figure 5). Identification of the full spectrum of relaxation times enables us to assess the impact of transient stretch on an isolated monolayer in the jammed state (figure 7). Noting that force regimes acting on epithelial tissue *in vivo* can vary widely, it is instructive to consider differences between fast and slow rates of stretch in terms of cell shape changes (leading to changes in cell pressure and tension) and cell stress (changing the isotropic cell stress *P*_eff, *i*_ and the cell shear stress *ζ*_i_). Our results reveal a heterogeneous response to homogeneous loading at intermediate loading rates, with peripheral cells showing the greatest relative change in tension under uniaxial loading and in shear stress under biaxial loading (figure 5). This distinct peripheral response is lost under fast stretch, and the response as a whole weakens in magnitude under slow stretch. In the future, it will be of interest to investigate how these findings relate to the biological mechano-responses (e.g. cell division) of epithelial tissue to stretch and whether these responses vary according to loading rate.

The vertex model is a gradient flow (appendix Ac), but not (typically) in a Euclidean metric. Nevertheless, motivated by Natale [33], it is instructive to compare the present model with a continuous gradient-flow model for a distribution function *ρ*(**x**, *t*) identifying vertex locations in ℝ^2^. For example, noting that cell areas are quadratic and symmetric in ***r*** (and ignoring tension effects temporarily), a kernel *K*(**x; y, z**, *t*) can be used to construct an area-like variable $A(x,t)=\frac{1}{2}\iint K(x;y,z,t)\rho (y,t)\rho (z,t)\text{d}y\;\text{d}z$. The dependence of *K* on **x** encodes the (potentially evolving) cellular microstructure and we impose *K*(**x; y, z**, *t*) = *K*(**x; z, y**, *t*). Using a suitable convex functional 𝒰[𝒜], which defines a pressure 𝒫(**x**, *t)* = *δ*𝒰/*δ*𝒜, one can construct an energy *U(t)* = ∫ 𝒰[𝒜(**x**, *t*)]d**x**, such that

(5.1) $$\frac{\delta U}{\delta \rho }(x;y,t)=𝒫(x,t)\frac{\delta A}{\delta \rho }(x;y,t),\;\frac{\delta A}{\delta \rho }(x;y,t)=\int K(x;y,z,t)\rho (z,t)\text{d}z.$$

Then, for some positive drag functional *E*[*ρ*] > 0, kinematics and the analogue of (2.7) give

(5.2) $$\rho_{t}+\nabla \cdot (\rho v)=0,\;E[\rho (y,t)]v(y,t)=-\nabla_{y}\int \frac{\delta U}{\delta \rho }(x;y,t)\text{d}x,$$

so that under suitable boundary conditions,

(5.3) $$\begin{array}{l} U_{t}=\iint 𝒫(x,t)\frac{\delta A}{\delta \rho }(x;y,t)\rho_{t}(y,t)\text{d}x\;\text{d}y=-\iint 𝒫(x,t)\frac{\delta A}{\delta \rho }(x;y,t)\nabla_{y}\cdot [\rho (y,t)v(y,t)]\text{d}x\;\text{d}y\\ \;=\iint \rho (y,t)v(y,t)\cdot \nabla_{y}\left[𝒫(x,t)\frac{\delta A}{\delta \rho }(x,y,t)\right]\text{d}x\;\text{d}y=-\int \rho E[\rho ]v\cdot v\;\text{d}y\leq 0, \end{array}$$

which recalls (2.11). The evolution (5.2) reduces to the system

(5.4a) $$\rho_{t}(y,t)=\int 𝒫(x,t)\nabla_{y}\cdot \left(\frac{\rho (y,t)}{E[\rho (y,t)]}\int \nabla_{y}K(x;y,z,t)\rho (z,t)\text{d}z\right)\text{d}x,$$

(5.4b) $$𝒫(x,t)=U^{\prime }\left[\frac{1}{2}\iint K(x;y,z,t)\rho (y,t)\rho (z,t)\text{d}y\text{d}z\right].$$

Additional terms could be included to account for tension effects. The resulting non-local nonlinear system falls into the broad category of aggregation–diffusion models [48], and suggests a route to a continuum representation of force chains [49], non-affine and long-range deformations [50,51] and related hyperbolic effects [52].

In summary, we have shown how SVD provides a natural framework with which to relate cell- and vertex-level dynamics in the relaxation of an isolated epithelial monolayer. Despite a clear role for scalar Laplacians (identifying 2*N*_*c*_ degrees of freedom related to material stiffness, driving primarily diffusive dynamics), simulations confirm the central role of geometric stiffness mediated by 2*N*_*v*_ degrees of freedom in determining the full relaxation spectrum. Considering the model as a gradient flow (e.g. (5.4a,b)), we have also shown how discrete approximations of some familiar spatial operators compete with more exotic non-local derivatives.

## Supplementary Material

Electronic supplementary material is available online at https://doi.org/10.6084/m9.figshare.c.7396505.

Appendix

## Data Availability

Simulation code is available from [44]. Supplementary material available online [47].
