# Supplementary material for: Spectral approaches to stress relaxation in epithelial monolayers
Source: Proc Math Phys Eng Sci. Author manuscript; Available in PMC 2026 Aug 20. (PMC7619384; doi:10.1098/rspa.2024.0224)
Supplement: Supplementary file 1 [file EMS217247-supplement-Appendix.pdf]

which recalls (2.11). The evolution (5.2) reduces to the system

$$\rho_t(\mathbf{y},t) = \int \mathcal{P}(\mathbf{x},t)\nabla_{\mathbf{y}} \cdot \left(\frac{\rho(\mathbf{y},t)}{E[\rho(\mathbf{y},t)]}\int \nabla_{\mathbf{y}} K(\mathbf{x};\mathbf{y},\mathbf{z},t)\rho(\mathbf{z},t)\,\mathrm{d}\mathbf{z}\right)\mathrm{d}\mathbf{x}, \tag{5.4a}$$

$$\mathcal{P}(\mathbf{x},t) = \mathcal{U}'\left[\frac{1}{2}\iint K(\mathbf{x};\mathbf{y},\mathbf{z},t)\rho(\mathbf{y},t)\rho(\mathbf{z},t)\mathrm{d}\mathbf{y}\mathrm{d}\mathbf{z}\right]. \tag{5.4b}$$

Additional terms could be included to account for tension effects. The resulting non-local nonlinear system falls into the broad category of aggregation–diffusion models [48], and suggests a route to a continuum representation of force chains [49], non-affine and long-range deformations [50,51] and related hyperbolic effects [52].

In summary, we have shown how SVD provides a natural framework with which to relate cell- and vertex-level dynamics in the relaxation of an isolated epithelial monolayer. Despite a clear role for scalar Laplacians (identifying $2N_c$ degrees of freedom related to material stiffness, driving primarily diffusive dynamics), simulations confirm the central role of geometric stiffness mediated by $2N_v$ degrees of freedom in determining the full relaxation spectrum. Considering the model as a gradient flow (e.g. (5.4a,b)), we have also shown how discrete approximations of some familiar spatial operators compete with more exotic non-local derivatives.

Data accessibility. Simulation code is available from [44].

Supplementary material available online [47].

Declaration of AI use. We have not used AI-assisted technologies in creating this article.

Authors' contributions. N.C.: data curation, formal analysis, investigation, methodology, software, visualization, writing—review and editing; C.K.R.: investigation, methodology, software, validation, writing—review and editing; E.J.: formal analysis, investigation, writing—review and editing; S.W.: funding acquisition, investigation, project administration, supervision, writing—review and editing; O.E.J.: conceptualization, formal analysis, funding acquisition, investigation, methodology, project administration, supervision, validation, writing—original draft, writing—review and editing.

All authors gave final approval for publication and agreed to be held accountable for the work performed therein.

Conflict of interest declaration. We declare we have no competing interests.

Funding. This work was supported by The Leverhulme Trust (RPG–2021–394), the Biotechnology and Biological Sciences Research Council (BB/T001984/1) and the Wellcome Trust (225408/Z/22/Z; 210062/Z/17/Z). For the purpose of open access, the authors have applied a Creative Commons Attribution (CCBY) licence to any Author Accepted Manuscript version arising.

Appendix A

(a) Geometric operators M and M' and the shape tensor Q

To calculate the geometric operators $\boldsymbol{M}$ and $\boldsymbol{M}'$, it is helpful to introduce signed incidence matrices A and B, which capture the topology of the cell network. We adopt notation used in Jensen & Revell and Jensen *et al*. [36,37]: $A_{jk} = 1$ (or -1) if directed cell edge j points into (out of) vertex k and is zero otherwise; $B_{ij} = 1$ (or -1) if directed cell edge j is congruent (anti-congruent) with the orientation of the face of cell i, and is zero otherwise. We define $\bar{A}_{jk} \equiv |A_{jk}|$ and $\bar{B}_{ij} \equiv |B_{ij}|$. All cell faces are assigned the same orientation $\boldsymbol{\epsilon}_i$, which is the 2×2 matrix representing a $\pi/2$ rotation; cell edge orientations are assigned arbitrarily. Edge vectors are then related to vertex locations by $\mathbf{t}_j = \sum_k A_{jk}\mathbf{r}_k$. Three useful identities are

$$\frac{\partial \mathbf{t}_j}{\partial \mathbf{r}_m} = A_{jm}\boldsymbol{I}_2, \quad \frac{\partial t_j}{\partial \mathbf{r}_m} = A_{jm}\check{\mathbf{t}}_j, \quad \frac{\partial \check{\mathbf{t}}_j}{\partial \mathbf{r}_m} = A_{jm}\frac{\boldsymbol{I}_2 - \check{\mathbf{t}}_j \otimes \check{\mathbf{t}}_j}{t_j}. \tag{A 1}$$

Downloaded from http://royalsocietypublishing.org/rspa/article-pdf/doi/10.1098/rspa.2024.0224/513231/rspa.2024.0224.pdf
by guest
on 29 July 2026

Here, a check denotes a unit vector, so that $\check{\mathbf{t}}_j = \mathbf{t}_j/t_j$. We define $\mathbf{n}_{ij} = -B_{ij}\epsilon_i\mathbf{t}_j$ to be the outward normal to cell i at edge j and $\mathbf{c}_j = \frac{1}{2}\sum_k \overline{A}_{jk}\mathbf{r}_k$ to be the centroid of edge j. The link $\mathbf{s}_{ik}$ in cell i between adjacent edge centroids at vertex k (figure 8*a*) and its normal $\mathbf{n}_{ik}$ pointing into the cell satisfy

$$\mathbf{s}_{ik} = \sum_j \tfrac{1}{2}B_{ij}\mathbf{t}_j\overline{A}_{jk}, \quad \mathbf{n}_{ik} \equiv \epsilon_i\mathbf{s}_{ik} = -\tfrac{1}{2}\sum_j \overline{A}_{jk}\mathbf{n}_{ij}\,. \tag{A 2}$$

Integrating over cell i with area A_i, $\boldsymbol{I}_2 A_i = \int_i \nabla\otimes\mathbf{x}\,\mathrm{d}A = \oint_i \check{\mathbf{n}}\otimes\mathbf{x}\,\mathrm{d}s = \sum_j \check{\mathbf{n}}_{ij}\otimes\oint\mathbf{x}\,\mathrm{d}s = \sum_j \mathbf{n}_{ij}\otimes\mathbf{c}_j = -\sum_j B_{ij}\epsilon_i\mathbf{t}_j\otimes\mathbf{c}_j$. Thus, small variations in area are related to small changes in vertex locations by

$$\boldsymbol{I}_2\,\delta A_i = \sum_{j,k}\left\{-\tfrac{1}{2}\sum_{k'} B_{ij}A_{jk}\overline{A}_{jk'}\epsilon_i\delta\mathbf{r}_k\otimes\mathbf{r}_{k'} - \tfrac{1}{2}B_{ij}\overline{A}_{jk}\epsilon_i\mathbf{t}_j\otimes\delta\mathbf{r}_k\right\}. \tag{A 3}$$

The identity

$$\sum_j B_{ij}A_{jk'}\bar{A}_{jk} = -\sum_j B_{ij}\bar{A}_{jk'}A_{jk}, \tag{A 4}$$

gives

$$\begin{aligned}\boldsymbol{I}_2\,\delta A_i &= \sum_{j,k}\left\{\frac{1}{2}\sum_{k'} B_{ij}\bar{A}_{jk}A_{jk'}\epsilon_i\delta\mathbf{r}_k\otimes\mathbf{r}_{k'} - \frac{1}{2}B_{ij}\bar{A}_{jk}\epsilon_i\mathbf{t}_j\otimes\delta\mathbf{r}_k\right\}\\ &= \sum_{j,k}\frac{1}{2}B_{ij}\bar{A}_{jk}\left\{\epsilon_i\delta\mathbf{r}_k\otimes\mathbf{t}_j - \epsilon_i\mathbf{t}_j\otimes\delta\mathbf{r}_k\right\}.\end{aligned} \tag{A 5}$$

Taking the trace,

$$2\delta A_i = \sum_j \tfrac{1}{2}B_{ij}\bar{A}_{jk}\left\{(\epsilon_i\delta\mathbf{r}_k)^T\mathbf{t}_j - (\epsilon_i\mathbf{t}_j)^T\delta\mathbf{r}_k\right\} = \sum_j \tfrac{1}{2}B_{ij}\bar{A}_{jk}\left\{-\delta\mathbf{r}_k^T\epsilon_i\mathbf{t}_j - \delta\mathbf{r}_k^T\epsilon_i\mathbf{t}_j\right\} \tag{A 6}$$

implying (using (A 2))

$$\frac{\partial A_i}{\partial\mathbf{r}_k} = -\tfrac{1}{2}\sum_j B_{ij}\overline{A}_{jk}\epsilon_i\mathbf{t}_j \equiv \tfrac{1}{2}\sum_j \overline{A}_{jk}\mathbf{n}_{ij} = -\mathbf{n}_{ik}\,. \tag{A 7a}$$

Noting that $L_i = \sum_j \bar{B}_{ij}t_j$, we use (A 1) to give

$$\frac{\partial L_i}{\partial\mathbf{r}_k} = \sum_j \overline{B}_{ij}A_{jk}\check{\mathbf{t}}_j\,. \tag{A 7b}$$

We use (A 7a,b) to construct the elements $\mathbf{M}_{\alpha k}$ of $\boldsymbol{M}$. It also allows us to express the vertex evolution equation (2.7) using incidence matrices as:

$$E_k\dot{\mathbf{r}}_k = \sum_{i,j}\left(\tfrac{1}{2}P_iB_{ij}\epsilon_i\mathbf{t}_j\bar{A}_{jk} - T_i\bar{B}_{ij}\check{\mathbf{t}}_jA_{jk}\right). \tag{A 8}$$

Cell stress is constructed from the first moments of $\boldsymbol{M}$, which, from (A 7a,b), are

$$\sum_k \mathbf{r}_k\otimes\frac{\partial A_i}{\partial\mathbf{r}_k} = \sum_{j,k}\tfrac{1}{2}\bar{A}_{jk}\mathbf{r}_k\mathbf{n}_{ij}^T = \sum_j \mathbf{c}_j\otimes\mathbf{n}_{ij} = \boldsymbol{I}_2A_i, \tag{A 9a}$$

$$\sum_k \mathbf{r}_k\otimes\frac{\partial L_i}{\partial\mathbf{r}_k} = \sum_{j,k}\overline{B}_{ij}A_{jk}\mathbf{r}_k\check{\mathbf{t}}_j^T = \sum_j \overline{B}_{ij}\mathbf{t}_j\otimes\check{\mathbf{t}}_j \equiv \boldsymbol{Q}_iL_i\,. \tag{A 9b}$$

The shape tensor $\boldsymbol{Q}_i$ is defined in (A 9b) and satisfies $\mathrm{Tr}(\boldsymbol{Q}_i) = 1$.
To build $\boldsymbol{M}'$, we differentiate (A 7a,b), giving

$$\frac{\partial^2 A_i}{\partial\mathbf{r}_k\partial\mathbf{r}_m} = -\frac{1}{2}\sum_j B_{ij}\bar{A}_{jk}A_{jm}\epsilon_i, \quad \frac{\partial^2 L_i}{\partial\mathbf{r}_k\partial\mathbf{r}_m} = \sum_j \bar{B}_{ij}A_{jk}A_{jm}\frac{\check{\mathbf{n}}_{ij}\otimes\check{\mathbf{n}}_{ij}}{t_j}, \tag{A 10}$$

noting that $\check{\mathbf{n}}_{ij}\otimes\check{\mathbf{n}}_{ij} = \boldsymbol{I}_2 - \check{\mathbf{t}}_j\otimes\check{\mathbf{t}}_j$ for cell i neighbouring edge j. It follows that $\partial^2 A_i/\partial\mathbf{r}_k^2 = \mathbf{0}$ (because $\mathsf{BA} = 0$). For vertices k neighbouring vertex m, with both neighbouring cell i and $m \neq k$,

Downloaded from http://royalsocietypublishing.org/rspa/article-pdf/doi/10.1098/rspa.2024.0224/513231/rspa.2024.0224.pdf
by guest
on 29 July 2026

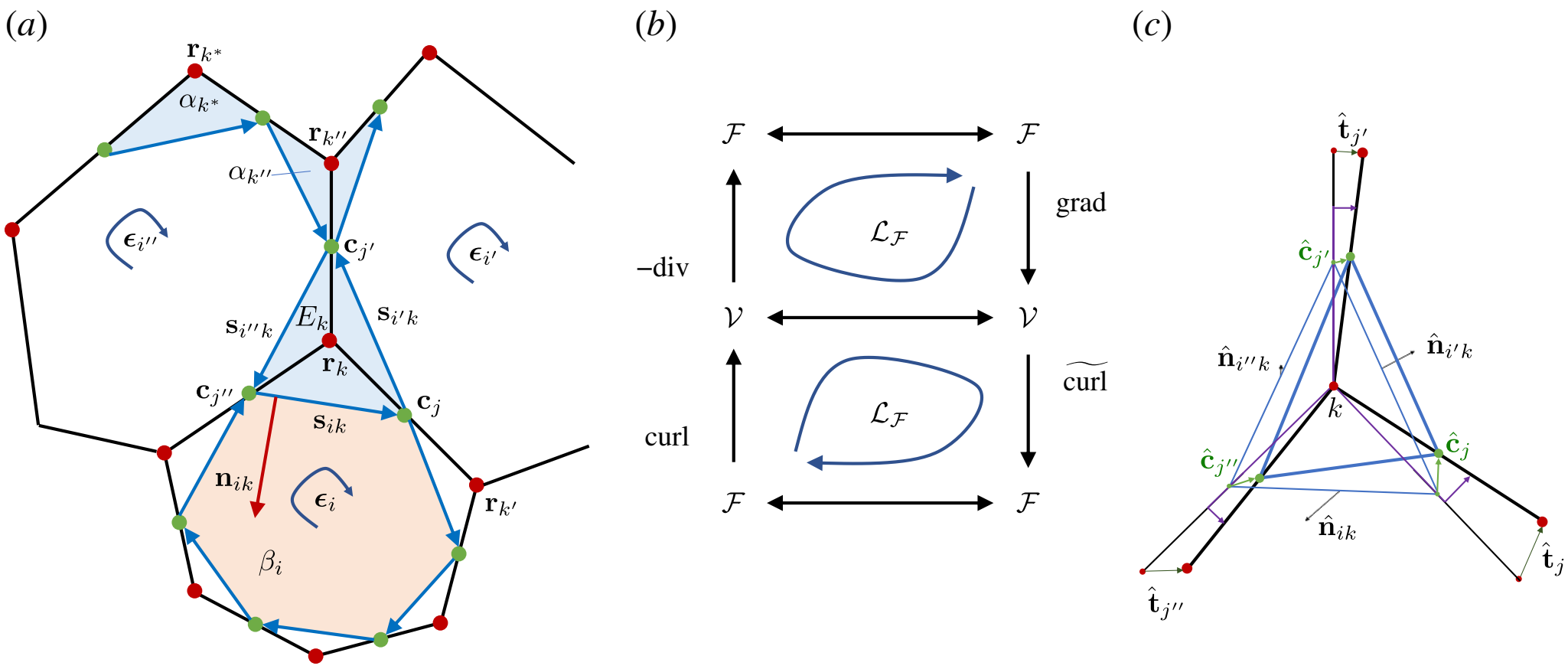

Figure 8. (*a*) Cell vertices (red circles) are connected by edges (black lines); edge centroids (green circles) are connected by links (blue lines) defined by (A 2) with normals $\mathbf{n}_{ik}$ defining $\partial A_i/\partial \mathbf{r}_k$ via (A 7a). Areas E_k around vertices and β_i within cells are shaded. Two types of peripheral vertex are illustrated, labelled as $\mathbf{r}_{k''}$ and $\mathbf{r}_{k^*}$; E_{k^*} and $E_{k''}$ are shaded. The orientation ϵ_i assigned to cell faces is here chosen to be clockwise in every cell. (*b*) Operators on the edge-centroid network. grad is defined by (A 13), its adjoint −div by (A 14) and the Laplacian over faces by (A 20). Curls are defined by (A 17). Exact sequences (A 19) are arranged vertically. (*c*) A small distortion of the network (from thin to thick edges) leads to changes $\hat{\mathbf{n}}_{ik}$ to the normals orthogonal to $\mathbf{s}_{ik}$, directed towards vertex k, along which pressures $\bar{P}_i$ act; changes to edges $\hat{\mathbf{t}}_j$ include rotation of unit vectors (purple arrows), along which tensions of adjacent cells $\bar{T}_i + \bar{T}_{i'}$ act.

$\partial^2 A_i/\partial \mathbf{r}_m \partial \mathbf{r}_k = \pm\frac{1}{2}\epsilon_i$, with values decreasing in the direction of ϵ_i; for such vertices, $\partial^2 L_i/\partial \mathbf{r}_k \partial \mathbf{r}_m$ takes the value $-\breve{\mathbf{n}}_{ij}\otimes\breve{\mathbf{n}}_{ij}/t_j$, where j is the edge connecting k and m.

(b) Discrete spatial derivatives

We consider how operators emerging from the vertex model relate to discrete approximations of conventional spatial derivatives over the network of links $\mathbf{s}_{ik}$ connecting adjacent edge centroids (see (A 2) and figure 8*a*). Following the scheme illustrated in figure 8*b*, we build the operators and their adjoints in terms of suitable inner products on $\mathcal{V}\subset\mathbb{R}^{N_v}\times\mathbb{R}^2$ (the vector space of vector-valued fields defined on vertices, equivalent to $\mathcal{TZ}$ in figure 1) and $\mathcal{F}\subset\mathbb{R}^{N_c}$ (the vector space of scalars defined on cell faces; this is distinct from $\mathcal{TY}$ in figure 1 in holding a single attribute per cell), such that for all $\boldsymbol{v}\in\mathcal{V}$, $\phi\in\mathcal{F}$ and $\psi\in\mathcal{F}$,

$$\langle \mathrm{grad}\ \phi, \boldsymbol{v}\rangle_{\mathsf{E}} = \langle\phi, -\mathrm{div}\ \boldsymbol{v}\rangle_{\mathcal{F}}, \quad \langle \mathrm{curl}\ \psi, \boldsymbol{v}\rangle_{\mathsf{E}} = \left\langle\psi, \widetilde{\mathrm{curl}}\ \boldsymbol{v}\right\rangle_{\mathcal{F}}. \tag{A 11}$$

$\langle\cdot,\cdot\rangle_{\mathsf{E}}$ is defined in (2.4a), in terms of the area E_k of the triangle at vertex k bounded by links between adjacent edge centroids (figure 8*a*). $\langle\cdot;\cdot\rangle_{\mathcal{F}}$ is equivalent to the component of $\langle\cdot,\cdot\rangle_{\mathsf{G}^{-1}}$ acting on pressure variations, so that $\langle \mathsf{f},\mathsf{g}\rangle_{\mathcal{F}} = \sum_i A_i f_i g_i$.

At internal vertex k, the three adjacent $\mathbf{s}_{ik}$ form a closed triangle, with vertices at the edge centroids $\mathbf{c}_j$, circulating in the orientation congruent to $\epsilon_k = -\epsilon_i$ (figure 8*a*). For fixed k, the vectors $\mathbf{n}_{ik} = \epsilon_i \mathbf{s}_{ik}$ form the three outward normals to the triangle surrounding vertex k. For the three edge centroid links shown in figure 8*a*, $\epsilon_i\mathbf{s}_{ik}\cdot\mathbf{s}_{i'k} = -2E_k$ and $\epsilon_i\mathbf{s}_{ik}\cdot\mathbf{s}_{i''k} = 2E_k$, which can be expressed more generally as

$$\epsilon_i\mathbf{s}_{ik}\cdot\mathbf{s}_{i'k} = 2E_k\sum_j A_{jk}B_{ij}\bar{B}_{i'j}. \tag{A 12}$$

Downloaded from http://royalsocietypublishing.org/rspa/article-pdf/doi/10.1098/rspa.2024.0224/513231/rspa.2024.0224.pdf
by guest
on 29 July 2026

Applying the identity [53] $\int \nabla\phi \, \mathrm{d}A = \oint \phi \check{\mathbf{t}} \times \check{\mathbf{n}} \, \mathrm{d}s$ to the triangle, we can define for a field $\phi \in \mathcal{F}$ the discrete operator grad approximating ∇ via

$$\{\text{grad } \phi\}_k = \frac{1}{E_k}\sum_i \epsilon_i \mathbf{s}_{ik}\phi_i = \frac{1}{E_k}\sum_{i,j} \frac{1}{2}\phi_i B_{ij}\epsilon_i \mathbf{t}_j \bar{A}_{jk} = -\frac{1}{E_k}\sum_i \frac{\partial A_i}{\partial \mathbf{r}_k}\phi_i, \tag{A 13}$$

using (A 7a). This shows how −grad P arises in the force balance (A 8).

From (A 11), the adjoint operator to grad is, for all $\boldsymbol{v} \in \mathcal{V}$,

$$\{-\text{div } \boldsymbol{v}\}_i = \frac{1}{A_i}\sum_k \epsilon_i \mathbf{s}_{ik} \cdot \mathbf{v}_k = -\frac{1}{A_i}\sum_k \frac{\partial A_i}{\partial \mathbf{r}_k} \cdot \mathbf{v}_k. \tag{A 14}$$

We can verify that div is exact for linear functions of position, namely that

$$\text{div } (\mathsf{K} \cdot \mathbf{r}_k + \mathbf{J}) = \text{Tr } (\mathsf{K}), \tag{A 15}$$

for a constant tensor K and vector **J**, as follows. $\sum_k -\epsilon_i \mathbf{s}_{ik} \otimes \mathbf{r}_k$ can be viewed as a line integral around the polygon within cell i bounded by links between edge centroids. Since $\mathbf{r}_k$ is constant along each link, we can write the outward normal to each link as the sum of outward normals to half-edges adjacent to vertex k using (A 2), making the integral one around the periphery of cell i, so that

$$-\sum_k \epsilon_i \mathbf{s}_{ik} \otimes \mathbf{r}_k = -\sum_{j,k} \tfrac{1}{2}\epsilon_i B_{ij}\mathbf{t}_j \bar{A}_{jk} \otimes \mathbf{r}_k = -\sum_j \tfrac{1}{2}\epsilon_i B_{ij}\mathbf{t}_j \otimes \mathbf{c}_j = \sum_j \mathbf{n}_{ij} \otimes \mathbf{c}_j = A_i \boldsymbol{I}_2, \tag{A 16}$$

where $\mathbf{n}_{ij}$ and $\mathbf{c}_j$ are as defined in appendix Aa. This ensures that div satisfies (A 15), so that div $\boldsymbol{r} = 2$ (which can be compared with (2.18)).

We define complementary curls around vertices and faces as

$$\{\text{curl f}\}_k = \frac{1}{E_k}\sum_i \mathbf{s}_{ik} f_i, \quad \left\{\widetilde{\text{curl}}\, \boldsymbol{v}\right\}_i = \frac{1}{A_i}\sum_k \mathbf{s}_{ik} \cdot \mathbf{v}_k. \tag{A 17}$$

Multiplying (A 16) by ϵ_i and taking the trace confirms that $\widetilde{\text{curl}}\, \boldsymbol{r} = 0$. To demonstrate that sequences in figure 8*b* are exact, the identity (A 12) implies that, when evaluating −div ∘ curl at cell i, the sum includes $-2f_{i'}$ from vertex k and $2f_{i'}$ from vertex k', leading to cancellation

$$\{-\text{div} \circ \text{curl f}\}_i = \frac{1}{A_i}\sum_{i',k} \frac{\epsilon_i \mathbf{s}_{ik} \cdot \mathbf{s}_{i'k}}{E_k} f_{i'} = \frac{2}{A_i}\sum_{i',j,k} A_{jk} B_{ij} \bar{B}_{i'j} f_{i'} = 0, \tag{A 18}$$

because $\sum_k A_{jk} = 0$. Thus,

$$-\text{div} \circ \text{curl} = 0, \quad \widetilde{\text{curl}} \circ \text{grad} = 0. \tag{A 19}$$

We can therefore define a scalar Laplacian over faces as

$$\{\mathcal{L}_{\mathcal{F}}\text{f}\}_i \equiv \{-\text{div} \circ \text{grad f}\}_i = \frac{1}{A_i}\sum_{i',k} \frac{(\epsilon_i \mathbf{s}_{ik}) \cdot (\epsilon_{i'} \mathbf{s}_{i'k})}{E_k} f_{i'} = \frac{1}{A_i}\sum_{i',k} \frac{\mathbf{s}_{ik} \cdot \mathbf{s}_{i'k}}{E_k} f_{i'}. \tag{A 20}$$

We recognize $\mathcal{L}_{\mathcal{F}}$ as $\mathsf{A}_c^{-1}\mathcal{L}_A$; recall $\mathcal{L}_A$ is the first block of $\mathcal{L}$ in (2.12) and therefore $\mathcal{L}_{\mathcal{F}}$ is the first block of $\mathsf{G}\mathcal{L} \equiv \mathcal{L}_c^{\mathsf{G}\dagger}$ in (2.13) and (3.10b). $\mathcal{L}_{\mathcal{F}}$ has the form $\mathsf{A}_c^{-1}\mathsf{BFB}^\top$, where $\mathsf{A}_c = \text{diag}(A_1, \ldots, A_{N_c})$ and F is diagonal, with elements defined along edges. Its spectrum is illustrated in the inset to figure 5*b*. Analogously, $\widetilde{\text{curl}} \circ \text{curl} = \mathcal{L}_{\mathcal{F}}$, as illustrated in figure 8*a*, while the associated vector Laplacian mapping $\mathcal{V} \to \mathcal{V}$ is $\boldsymbol{\mathcal{L}}_{\mathcal{F}} \equiv -\text{grad} \circ \text{div} + \text{curl} \circ \widetilde{\text{curl}}$. This system also admits a set of dual operators mapping scalars defined over vertices to vectors defined over faces.

Downloaded from http://royalsocietypublishing.org/rspa/article-pdf/doi/10.1098/rspa.2024.0224/513231/rspa.2024.0224.pdf
by guest
on 29 July 2026

royalsocietypublishing.org/journal/rspa *Proc. R. Soc. A* **480**: 20240224

Having identified the force owing to pressure on a vertex with grad P, where grad mimics ∇, let us consider the corresponding force owing to tension in (A 8). We define a new gradient operator mapping scalar fields on cells to vector fields on vertices,

$$\{\text{grad}\ \mathsf{T}\}_k = -\frac{1}{E_k}\sum_j \check{\mathbf{t}}_j A_{jk}\bar{B}_{ij}T_i\,. \tag{A 21}$$

This gives the vector sum of tensions acting along each edge radiating outward from vertex k. Now defining $\langle \mathsf{f},\mathsf{g}\rangle_{\mathcal{F}} \equiv \sum_j L_i f_i g_i$ (consistent with (2.3)), then under inner products $\langle\cdot,\cdot\rangle_{\mathsf{E}}$ and $\langle\cdot,\cdot\rangle_{\mathcal{F}}$ we can define the adjoint operator as

$$\{-\,\text{div}\ \mathbf{v}\}_i = -\tfrac{1}{L_i}\sum_{j,k}\bar{B}_{ij}A_{jk}\check{\mathbf{t}}_j\cdot\mathbf{v}_k\,. \tag{A 22}$$

Summing over the vertices k of cell i, this sums components of $\mathbf{v}_k$ pointing outwards from the cell. The operator −div ∘ grad is a scalar Laplacian with element ii' being

$$\tfrac{1}{L_i}\sum_{j,j',k}\bar{B}_{ij}A_{jk}\frac{\check{\mathbf{t}}_j\cdot\check{\mathbf{t}}_{j'}}{E_k}A_{j'k}\bar{B}_{i'j'}\,. \tag{A 23}$$

We recognize (A 23) as $\mathsf{L}_c^{-1}\mathcal{L}_L$, where $\mathcal{L}_L$ is the final block of $\mathcal{L}$ in (2.12), making (A 23) equivalent to the final block of $\mathsf{G}\mathcal{L} \equiv \mathcal{L}_c^{\mathsf{G}\dagger}$ in (2.13) and (3.10b). Figure 5*b* shows how the spectrum of (A 23) captures cell-dilation modes when Γ is large.

(c) Gradient flow formulation

Here, we follow formalism presented by Peletier [54]. The state space $\mathcal{Z} = \mathbb{R}^{N_v}\times\mathbb{R}^2$ has elements $\boldsymbol{r}(t)$ (in the sense that the evolution at any instant is determined entirely by vertex locations, for a given network topology). Its tangent space $\mathcal{TZ}$ at $\boldsymbol{r}$ has elements $\boldsymbol{v}(t)$, representing vertex velocities, so that $\dot{\boldsymbol{r}}\in\mathcal{TZ}$. The energy U is a map from $\mathcal{Z}$ to $\mathbb{R}$, when we consider $U = U(\boldsymbol{r})$. The (dual) cotangent space $\mathcal{TW}$ has elements $\boldsymbol{f}$ representing forces on vertices; we combine elements of the tangent and cotangent spaces via the scalar product (a bilinear form) $\boldsymbol{f}^{\top}\cdot\boldsymbol{v}\equiv\boldsymbol{v}^{\top}\cdot\boldsymbol{f}$. The Fréchet derivative U_r of the energy with respect to vertex displacements is defined via

$$\lim_{s\to 0}\frac{U(\boldsymbol{r}+s\boldsymbol{v})-U(\boldsymbol{r})}{s} = U_{\boldsymbol{r}}^{\top}\cdot\boldsymbol{v}\quad \text{for all}\quad \boldsymbol{v}\in\mathcal{TZ}\,. \tag{A 24}$$

Thus, $U\boldsymbol{r}(\boldsymbol{r})\equiv\boldsymbol{M}^{\top}\mathsf{g}\in\mathcal{TW}$ and $\dot{U}\equiv(U_r(\boldsymbol{r}))^{\top}\cdot\dot{\boldsymbol{r}} = \left(\boldsymbol{M}^{\top}\mathsf{g}\right)^{\top}\cdot\dot{\boldsymbol{r}} = \mathsf{g}^{\top}\boldsymbol{M}\cdot\dot{\boldsymbol{r}}$.

We write the evolution (2.10) in canonical form as $\mathcal{D}(\boldsymbol{r})\dot{\boldsymbol{r}} = -\,U_r(\boldsymbol{r})$. The dissipation operator $\mathcal{D}$ maps elements of $\mathcal{TZ}$ to elements of $\mathcal{TW}$. Defining the bilinear form

$$(\boldsymbol{v}_1,\boldsymbol{v}_2)_{\mathcal{D},r} = \boldsymbol{v}_1^{\top}\cdot\left(\mathsf{E}\otimes\mathsf{I}_2+\boldsymbol{M}^{\top}\mathsf{H}\boldsymbol{M}\right)\cdot\boldsymbol{v}_2, \tag{A 25}$$

we can write (2.11) as $\dot{U} = -\,(\dot{\boldsymbol{r}},\dot{\boldsymbol{r}})_{\mathcal{D},r}\le 0$. Treating (A 25) as an inner product (it is symmetric, bilinear and positive definite), we can write (2.10) as $\dot{\boldsymbol{r}} = -\,\text{grad}_{\mathcal{D}}\,U\in\mathcal{TZ}$. In other words,

$$(\boldsymbol{v},\dot{\boldsymbol{r}})_{\mathcal{D},r} = -\,(\boldsymbol{v},\text{grad}_{\mathcal{D}}\,U)_{\mathcal{D},r}\equiv -\,\boldsymbol{v}^{\top}\cdot U_{\boldsymbol{r}}\quad\text{for all}\quad \boldsymbol{v}\in\mathcal{TZ}\,. \tag{A 26}$$

This definition of $\text{grad}_{\mathcal{D}}U$ shows how it is the Riesz representation of the Fréchet derivative U_r, and is determined by the dissipation inner product.

Downloaded from http://royalsocietypublishing.org/rspa/article-pdf/doi/10.1098/rspa.2024.0224/513231/rspa.2024.0224.pdf
by guest
on 29 July 2026

(d) Distinguishing cell-shear and cell-dilation modes

We write (2.13) in block form as

$$\begin{pmatrix} \mathsf{I}_{N_c} + \gamma_A \mathsf{A}_c^{-1}\mathcal{L}_A \mathsf{A}_c & \mathsf{A}_c^{-1}\mathcal{L}_C \mathsf{L}_c(\gamma_L/\Gamma L_0) \\ \mathsf{L}_c^{-1}\mathcal{L}_C^{\top}\mathsf{A}_c\gamma_A \Gamma L_0 & \mathsf{I}_{N_c} + \gamma_L \mathsf{L}_c^{-1}\mathcal{L}_L \mathsf{L}_c \end{pmatrix} \begin{pmatrix} \dot{\mathsf{P}} \\ \dot{\mathsf{T}} \end{pmatrix} = - \begin{pmatrix} \mathsf{A}_c^{-1}\mathcal{L}_A & \mathsf{A}_c^{-1}\mathcal{L}_C \\ \Gamma L_0 \mathsf{L}_c^{-1}\mathcal{L}_C^{\top} & \Gamma L_0 \mathsf{L}_c^{-1}\mathcal{L}_L \end{pmatrix} \begin{pmatrix} \mathsf{P} \\ \mathsf{T} \end{pmatrix}. \tag{A 27}$$

For $\Gamma \sim \gamma_L \ll 1$, cell-dilation modes are recovered by setting $\mathsf{T} = \Gamma \hat{\mathsf{T}}$ in (A 27) and discarding terms of $O(\Gamma)$, to give (2.14a) with $\mathsf{T} \approx \Gamma L_0 \mathsf{L}_c^{-1}\mathcal{L}_C^{\top}\mathcal{L}_A^{-1}\mathsf{A}_c\mathsf{P}$ (tensions are weak and are slaved to pressures). Slower cell-shear modes are recovered by setting $t = \tau/\Gamma$ in (A 27), to give (at leading order in Γ) strong coupling between pressure and tension (but with opposite parity to cell-dilation modes), $\mathsf{P} \approx -\mathcal{L}_A^{-1}\mathcal{L}_C\mathsf{T}$, with $\dot{\mathsf{T}} \approx -\Gamma L_0 \mathsf{L}_c^{-1}(\mathcal{L}_L - \mathcal{L}_C^{\top}\mathcal{L}_A^{-1}\mathcal{L}_C)\mathsf{T}$ (that is, the modes have the spectrum of the Schur complement $\mathsf{G}\mathcal{L}/(\mathsf{A}_c^{-1}\mathcal{L}_A)$).

For $\Gamma \sim \gamma_A^{-1} \gg 1$, cell-dilation modes are recovered by setting $t = \tau/\Gamma$ and $\mathsf{P} = \hat{\mathsf{P}}/\Gamma$ in (A 27), to give (2.14b) at leading order in $1/\Gamma$ with $\mathsf{P} \approx \mathsf{A}_c^{-1}\mathcal{L}_C\mathcal{L}_L^{-1}\mathsf{L}_c\mathsf{T}/(\Gamma_0 L_0)$ (now, pressures are weak and are slaved to tensions). Slower cell-shear modes are recovered by taking dominant terms in (A 27) for large Γ, giving $\mathsf{T} \approx -\mathcal{L}_L^{-1}\mathcal{L}_C^{\top}\mathsf{P}$ (strong coupling, with opposite parity to cell-dilation modes) with $\dot{\mathsf{P}} \approx -\mathsf{A}_c^{-1}(\mathcal{L}_A - \mathcal{L}_C\mathcal{L}_L^{-1}\mathcal{L}_C^{\top})\mathsf{P}$ (the spectrum is that of the Schur complement $\mathsf{G}\mathcal{L}/(\Gamma L_0 \mathsf{L}_c^{-1}\mathcal{L}_L)$).

(e) Connecting prestress to stiffness

Referring to figure 8*c*, (2.20a) and (A 10), we can write the restoring force exerted on vertex k by network prestress, under a small distortion of the network, as

$$-\left\{\bar{\mathsf{E}}^{-1}\bar{\mathsf{g}}^{\top}\bar{\boldsymbol{M}}' \cdot \hat{\boldsymbol{r}}\right\}_k = -\frac{1}{\bar{E}_k}\left[\bar{P}_i\widehat{\mathbf{n}}_{ik} + \bar{P}_{i'}\widehat{\mathbf{n}}_{i'k} + \bar{P}_{i''}\widehat{\mathbf{n}}_{i''k}\right]$$
$$-\check{\mathbf{n}}_{ij}\frac{\check{\mathbf{n}}_{ij}\cdot\hat{\mathbf{t}}_j}{t_j}[\bar{T}_i + \bar{T}_{i'}] - \check{\mathbf{n}}_{i'j'}\frac{\check{\mathbf{n}}_{i'j'}\cdot\hat{\mathbf{t}}_{j'}}{t_{j'}}[\bar{T}_{i'} + \bar{T}_{i''}] - \check{\mathbf{n}}_{i''j''}\frac{\check{\mathbf{n}}_{i''j}\cdot\hat{\mathbf{t}}_{j''}}{t_{j''}}[\bar{T}_{i''} + \bar{T}_i]\,. \tag{A 28}$$

Recall $\mathbf{n}_{ik}$ is normal to $\mathbf{s}_{ik}$, the link in cell i between adjacent edge centroids at vertex k, satisfying (A 6). Pressure of cell i on vertex k acts along $\mathbf{n}_{ik} = \boldsymbol{\epsilon}_i\mathbf{s}_{ik}$; distortion of the network changes the orientation of the normal as indicated by $\hat{\mathbf{n}}_{ik}$. The distorted normals sum to zero $(\sum_i \hat{\mathbf{n}}_{ik} = 0)$, indicating that the restoring force owing to pressure vanishes if $\bar{P}_i = \bar{P}_{i'} = \bar{P}_{i''}$. An alternative expression for the pressure terms in (A 26) is, using (A 4),

$$\frac{1}{E_k}\boldsymbol{\epsilon}_i\left[(\bar{P}_{i'} - \bar{P}_i)\hat{\mathbf{c}}_j + (\bar{P}_{i''} - \bar{P}_{i'})\hat{\mathbf{c}}_{j'} + (\bar{P}_i - \bar{P}_{i''})\hat{\mathbf{c}}_{j''}\right] \tag{A 29}$$

showing how pressure differences between cells act in the direction orthogonal to edge centroid displacements. Tensions of the cells i and i' adjacent to edge j act together along the unit vector pointing away from vertex k; distortion of the network rotates the unit vector, generating a force orthogonal to edge j.

(f) Stretch

Consider a monolayer sitting on a membrane that undergoes a prescribed deformation. We model adhesion between cells and the membrane by modifying the drag at vertices. In a reference configuration, suppose the monolayer and membrane are in equilibrium with vertex locations $\mathbf{r}_k$. These identify locations $\mathbf{R}_k$ on the membrane. The membrane then undergoes

Downloaded from http://royalsocietypublishing.org/rspa/article-pdf/doi/10.1098/rspa.2024.0224/513231/rspa.2024.0224.pdf
by guest
on 29 July 2026

biaxial stretch, mapping $\boldsymbol{R}$ to $\boldsymbol{\Lambda}\cdot\boldsymbol{R}$, where $\boldsymbol{\Lambda}\equiv \mathsf{I}_{N_v}\otimes\left(\Lambda_1(t)\mathbf{e}_1\mathbf{e}_1^T+\Lambda_2(t)\mathbf{e}_2\mathbf{e}_2^T\right)$. Here, $\mathbf{e}_1$ and $\mathbf{e}_2$ are fixed orthogonal axes in the plane of the membrane and Λ_1 and Λ_2 are stretches. The force balance (2.7) then becomes $\mathsf{E}(\dot{\boldsymbol{r}}-\dot{\boldsymbol{\Lambda}}\cdot\boldsymbol{R})=-\boldsymbol{M}^{\mathsf{T}}\mathsf{g}$, penalizing increasing displacements between $\boldsymbol{r}$ and $\boldsymbol{\Lambda}\cdot\boldsymbol{R}$, dragging each vertex towards its counterpart on the membrane whenever there is membrane motion. In this model, rapid membrane stretching promotes strong deformation of the monolayer. Assuming that the membrane does not interfere with dissipation associated with area and perimeter changes, this leads to a forcing term $\mathsf{E}\dot{\boldsymbol{\Lambda}}\cdot\boldsymbol{R}$ on the right-hand side of (2.10). Areas and perimeters then evolve according to a modified form of (2.12),

$$\left(\mathsf{I}_{2N_c}+\mathcal{L}\mathsf{H}\right)\dot{\mathsf{s}}=-\mathcal{L}\mathsf{g}+\boldsymbol{M}\cdot\dot{\boldsymbol{\Lambda}}\cdot\boldsymbol{R},\quad \mathsf{g}=\mathcal{G}(\mathsf{s};\mathsf{s}_0,\Gamma)\,. \tag{A 30}$$

In figure 7, we impose $\dot{\Lambda}_1=\pm\dot{\Lambda}_2=10^{-2}$ for $0<t<\tau$, and zero thereafter, for uniaxial (−) and biaxial (+) stretch.

References

1. Nagai T, Honda H. 2001 A dynamic cell model for the formation of epithelial tissues. *Philos. Mag. B.* **81**, 699–719. (doi:10.1080/13642810108205772)
2. Farhadifar R, Röper JC, Aigouy B, Eaton S, Jülicher F. 2007 The influence of cell mechanics, cell-cell interactions, and proliferation on epithelial packing. *Curr. Biol.* **17**, 2095–2104. (doi:10.1016/j.cub.2007.11.049)
3. Alt S, Ganguly P, Salbreux G. 2017 Vertex models: from cell mechanics to tissue morphogenesis. *Phil. Trans. R. Soc. B* **372**, 20150520. (doi:10.1098/rstb.2015.0520)
4. Fletcher AG, Cooper F, Baker RE. 2017 Mechanocellular models of epithelial morphogenesis. *Phil. Trans. R. Soc. B* **372**, 20150519. (doi:10.1098/rstb.2015.0519)
5. Guillot C, Lecuit T. 2013 Mechanics of epithelial tissue homeostasis and morphogenesis. *Science* **340**, 1185–1189. (doi:10.1126/science.1235249)
6. Nestor-Bergmann A, Johns E, Woolner S, Jensen OE. 2018 Mechanical characterization of disordered and anisotropic cellular monolayers. *Phys. Rev. E.* **97**, 052409. (doi:10.1103/PhysRevE.97.052409)
7. Tong S, Singh NK, Sknepnek R, Košmrlj A. 2022 Linear viscoelastic properties of the vertex model for epithelial tissues. *PLoS Comput. Biol.* **18**, e1010135. (doi:10.1371/journal.pcbi.1010135)
8. Bi D, Lopez JH, Schwarz JM, Manning ML. 2015 A density-independent rigidity transition in biological tissues. *Nat. Phys.* **11**, 1074–1079. (doi:10.1038/nphys3471)
9. Koride S, Loza AJ, Sun SX. 2018 Epithelial vertex models with active biochemical regulation of contractility can explain organized collective cell motility. *APL Bioeng.* **2**, 031906. (doi:10.1063/1.5023410)
10. Brodland GW, Veldhuis JH, Kim S, Perrone M, Mashburn D, Hutson MS. 2014 Cellfit: a cellular force-inference toolkit using curvilinear cell boundaries. *PLoS One* **9**, e99116. (doi:10.1371/journal.pone.0099116)
11. Kursawe J, Baker RE, Fletcher AG. 2018 Approximate Bayesian computation reveals the importance of repeated measurements for parameterising cell-based models of growing tissues. *J. Theor. Biol.* **443**, 66–81. (doi:10.1016/j.jtbi.2018.01.020)
12. Noll N, Streichan SJ, Shraiman BI. 2020 Variational method for image-based inference of internal stress in epithelial tissues. *Phys. Rev. X.* **10**, 011072. (doi:10.1103/physrevx.10.011072)
13. Roffay C, Chan CJ, Guirao B, Hiiragi T, Graner F. 2021 Inferring cell junction tension and pressure from cell geometry. *Development* **148**, dev192773. (doi:10.1242/dev.192773)
14. Ogita G, Kondo T, Ikawa K, Uemura T, Ishihara S, Sugimura K. 2022 Image-based parameter inference for epithelial mechanics. *PLoS Comput. Biol.* **18**, e1010209. (doi:10.1371/journal.pcbi.1010209)

royalsocietypublishing.org/journal/rspa *Proc. R. Soc. A* **480**: 20240224

Downloaded from http://royalsocietypublishing.org/rspa/article-pdf/doi/10.1098/rspa.2024.0224/513231/rspa.2024.0224.pdf
by guest
on 29 July 2026
